# Redox and Photochemical Reactivity of Cerium(IV) Carbonate and Carboxylate Complexes Supported by a Tripodal Oxygen Ligand

**DOI:** 10.1021/acs.inorgchem.4c04602

**Published:** 2025-01-15

**Authors:** Hoang-Long Pham, Xinxin Jiang, Qiaolin Yan, Yat-Ming So, Wan Chan, Herman H. Y. Sung, Ian D. Williams, Wa-Hung Leung

**Affiliations:** Department of Chemistry, The Hong Kong University of Science and Technology, Clear Water Bay, Kowloon, Hong Kong 999077, China

## Abstract

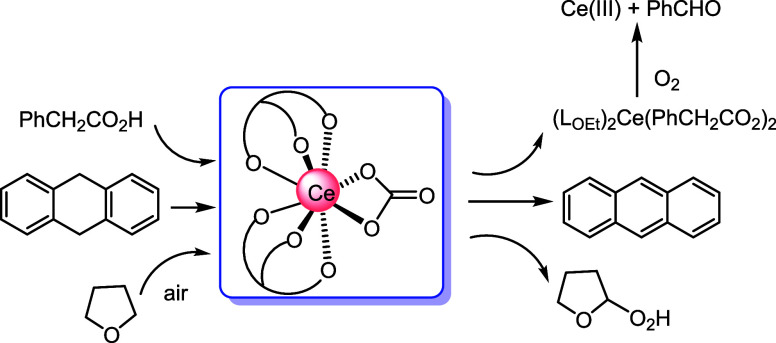

The protonolysis and redox reactivity of a Ce(IV) carbonate complex supported by the Kläui tripodal ligand [(η^5^-C_5_H_5_)Co{P(O)(OEt)_2_}_3_]^−^ (L_OEt_^–^) have been studied. Whereas treatment of [Ce(L_OEt_)_2_(CO_3_)] (**1**) with RCO_2_H afforded [Ce(L_OEt_)_2_(RCO_2_)_2_] (*R* = Me (**2**), Ph (**3**), 2-NO_2_C_6_H_3_ (**4**)), the reaction of **1** with PhCH_2_CO_2_H resulted in formation of a mixture of Ce(IV) (**5**) and Ce(III) (**6**) carboxylate species. In benzene in the dark, **5** was slowly converted into **6** via Ce(IV)-O(carboxylate) homolysis. Recrystallization of a mixture of **5** and **6** from hexane led to isolation of yellow crystals of **6** that were identified as [Ce(L_OEt_)_2_(PhCH_2_CO_2_)]. Treatment of **1** with sulfamic acid, trifluoroacetamide, and trifluoromethanesulfonamide gave [Ce(L_OEt_)_2_(SO_3_NH)_2_] (**7**), [Ce(L_OEt_)_2_(CF_3_CONH)_2_] (**8**), and [Ce(L_OEt_)_2_(CF_3_SO_2_NH)_2_] (**9**), respectively. The crystal structures of **2**, **4**, and **6–8** have been determined. H atom transfer (HAT) of 2,6-di-*tert*-butylphenol and 9,10-dihydroanthracene (DHA) with **1** afforded 3,3′,5,5′-tetra-*tert*-butyldiphenoquinone and anthracene, respectively. The oxidation of DHA with **1** under air yielded anthracene and anthraquinone. While **1** is stable in acetonitrile, it is readily reduced to a Ce(III) species in tetrahydrofuran. In air, **1** reacted with tetrahydrofuran to produce tetrahydrofuran hydroperoxide that can reduce the Ce(IV) carbonate rapidly. Upon irradiation with blue LED light, the Ce(IV)-L_OEt_ carboxylate complexes underwent facile decarboxylation via Ce–O homolysis. **1** proved to be an efficient catalyst precursor for decarboxylative oxygenation of arylacetic acids. For example, irradiation of phenylacetic acid with blue LED light in the presence of 5 mol % of **1** under air afforded benzyl alcohol and benzaldehyde in 10 and 90% yield, respectively.

## Abstract 2

Protonolysis of the Ce(IV) carbonate [Ce(L_OEt_)_2_(CO_3_)] (L_OEt_^−^ = [CpCo{P(O)(OEt)_2_}_3_]^−^) (**1**) with carboxylic acids and other acidic compounds has been studied. **1** can oxidize substituted phenols, 9,10-dihydroanthracene, and tetrahydrofuran via H-atom transfer. **1** has been used as a catalyst precursor for photodecarboxylative oxygenation of arylacetic acids.

## Introduction

Although a few molecular terbium(IV) and praseodymium(IV) complexes supported by electron-rich ligands have been isolated recently,^1^ cerium is the only lanthanide element that can form stable tetravalent complexes in solution.^2^ Many applications of Ce(IV) compounds, notably ceric ammonium nitrate (CAN),^3^ is related to the oxidizing power of Ce(IV). Recent studies have shown that ligands have an pronounced influence on the redox behavior of Ce(IV) complexes.^4^ Nevertheless, despite the widespread use of Ce(IV) reagents in organic oxidations, the role of ligands in the oxidation chemistry of molecular Ce(IV) complexes is not well understood.

Carbonate is a versatile ligand that can bind to metal ions in various coordination modes.^5^ Molecular lanthanide carbonate complexes such as [Ln(CO_3_)_n_]^(2n-3)–^ are well-known.^6^ Polymeric lanthanide carbonate materials have attracted current attention owing to their interesting luminescent properties and their use as precursors to nanomaterials.^7^ Moreover, dianionic carbonate is an excellent supporting ligand of high-valent metal ions. Some transition metal (e.g., Cu, Ni, Cu) carbonates are active catalysts for electrochemical water oxidation, in which high-valent metal carbonate species have been provoked to be active intermediates.^8,9,10,11,12^ Although molecular Ce(IV) carbonate compounds have been known for a long time,^13,14^ the reactivity of the coordinated carbonate ligand at the Ce(IV) center has not been well explored.

**Scheme 1:**
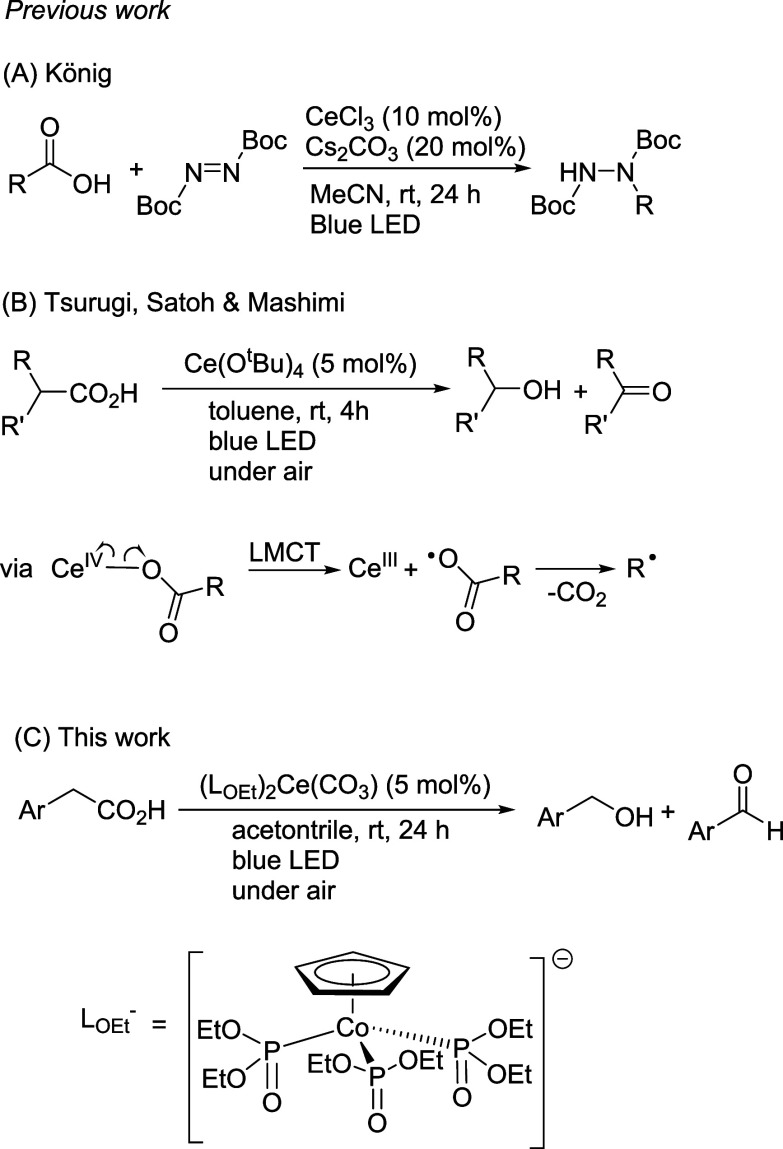
Examples of Cerium-Catalyzed Decarboxylation of Carboxylic Acids

Recently, we have demonstrated that Ce(IV) alkoxide^15^ and aryloxide^16^ complexes supported by the Kläui ligand [Co(η^5^-C_5_H_5_){P(O)(OEt)_2_}_3_]^−^ (L_OEt_^–^), [Ce(L_OEt_)_2_(OR)_2_] (*R* = alkyl or aryl), can oxidize substituted phenols and 9,10-dihydroanthracene (DHA) via proton-coupled electron transfer (PCET). The ability of the Ce(IV) alkoxides and aryloxides to oxidize X–H (X = O, C) bonds is attributed to the cooperativity of the oxidizing Ce^4+^ ion and the basic alkoxide/aryloxide ligand. In this context, Ce(IV) complexes with basic carbonate ligands are expected to be a good platform for studying Ce-based PCET oxidations. In this paper, the reactivity of the Ce(IV) carbonate complex [Ce(L_OEt_)_2_(CO_3_)] (Scheme 1)^17^ toward organic compounds bearing acidic/reactive X–H bonds, including carboxylic acids, acetamide, sulfonamide, phenols, and DHA, has been studied.

It is well-known that Ce(IV) carboxylates are light-sensitive and prone to photoreduction. Sheldon and Kochi first reported that photolysis of Ce(IV) alkylcarboxylates with UV light resulted in Ce–O homolysis and formation of alkyl radicals.^18^ Recently, Ce-mediated photodecarboxylation has been utilized in organic synthesis.^19,20,21^ For instance, König and coworkers reported that CeCl_3_ in conjunction with Cs_2_CO_3_ can catalyze the photodecarboxylative hydrazination of carboxylic acids via Ce–O homolysis of Ce(IV) carboxylate intermediates (Scheme 1A).^19^ Tsurugi, Satoh, Mashima and coworkers have shown that Ce(IV) carboxylate clusters derived from [Ce(O^t^Bu)_4_] can function as photocatalysts for decarboxylative oxygenation (Scheme 1B) and lactonization of carboxylic acids through ligand-to-metal charge-transfer (LMCT) activation of the Ce(IV)-O_2_CR chromophore.^20^ In this work, we found that [Ce(L_OEt_)_2_(RCO_2_)_2_] underwent facile decarboxylation via Ce–O(carboxylate) homolysis under mild conditions. As Ce(IV) carboxylates are easily accessed via protonolysis of the carbonate precursor, **1** has been used as a convenient, air-stable precatalyst for decarboxylative oxygenation of arylacetic acids (Scheme 1C).

## Results and Discussion

### Carboxylate Complexes

**Scheme 2:**
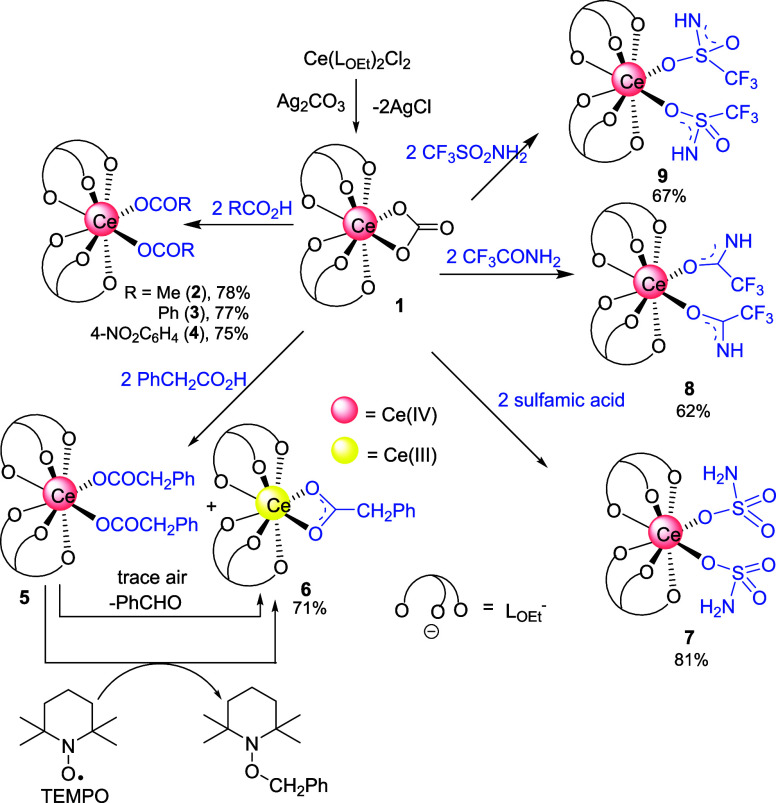
Protonolysis of 1

Previously, we synthesized a Ce(IV) carbonate complex, [Ce(L_OEt_)_2_(CO_3_)] (**1**), via 1,2-insertion of CO_2_ with a Ce(IV) oxo complex, [Ce(L_OEt_)_2_(O)(H_2_O)]·MeCONH_2_. The structure of **1** featuring a bidentate carbonate ligand has been established by X-ray diffraction.^17^ In this work, we found that **1** could be synthesized conveniently by salt metathesis of [Ce(L_OEt_)_2_Cl_2_]^17a^ with Ag_2_CO_3_. Owing to the basicity of the carbonate ligand, **1** underwent protonolysis readily with acidic compounds such as carboxylic acids (Scheme 2). For example, treatment of **1** with acetic acid and benzoic acid in MeCN afforded the Ce(IV) bis(carboxylate) complexes [Ce(L_OEt_)_2_(RCO_2_)_2_] (*R* = Me (**2**), Ph (**3**)). The crude products of **2** and **3** were found to contain small amounts of Ce(III) impurities that could be removed by recrystallization from hexane. In previous work, the acetate **2** has been synthesized via acylation of [Ce(L_OEt_)_2_(O)(H_2_O)]·MeCONH_2_ with acetic anhydride.^17b^ The ^1^H NMR spectrum of **3** displayed sharp signals due to the L_OEt_^–^ and benzoate ligands in a 1:1 ratio, which is consistent with its formula. The ^31^P NMR resonance of **3** occurred at δ 116.28 ppm that is typical for diamagnetic Ce(IV)-L_OEt_ complexes.^22^ Despite several attempts, we have not been able to obtain single crystals of **3** for structure determination. However, we have structurally characterized the 2-nitrobenzoate analogue [(L_OEt_)_2_Ce(2-NO_2_C_6_H_4_CO_2_)_2_] (**4**) that was synthesized from **1** and 2-nitrobenzoic acid.

On the other hand, the protonolysis of **1** with phenylacetic acid in MeCN in the dark led to isolation of a ca. 3:1 mixture of a Ce(IV) (**5**) and a Ce(III) (**6**) species that exhibited ^31^P NMR resonances at δ 119.75 and 153.84 ppm, respectively. Attempts to reduce the formation of the Ce(III) product by shortening the reaction time or using a different solvent failed. Furthermore, **5** was found to be converted into **6** in solution, rendering the isolation of a pure sample of **5** difficult. In benzene-*d*_6_ in the dark, the **5**:**6** ratio of the mixture dropped from 3:1 to 1:2 in 3 h (Figure S10, Supporting Information), and concomitantly benzaldehyde that was identified by GC-MS was produced, indicating that the conversion of **5** into **6** involved decarboxylation of the phenylacetate ligand, followed by oxidation of the generated benzyl radical with adventitious oxygen. The formation of benzyl radical has been confirmed by a radical trap experiment. Thus, addition of (2,2,6,6-tetramethylpiperidin-1-yl)oxyl (TEMPO) to a mixture of **5**:**6** in benzene resulted in formation of TEMPO–CH_2_Ph that was identified by LC-MS. By contrast, both isolated **2** and **3** are stable in benzene in the dark under the same conditions. Although photoreduction of Ce(IV) carboxylates is well documented,^18,20^**5** is a rare example of a metal carboxylate complex that undergoes nonphotochemical decarboxylation at room temperature.^23^ The facile decarboxylation of **5** can be attributed to the stability of the generated benzyl radical and/or multiconfigurational nature of the Ce(IV) complex.^16,24,25^

Recrystallization of a mixture of **5** and **6** from hexane for 3 d led to isolation of yellow single crystals of **6** that were identified as [Ce(L_OEt_)_2_(PhCH_2_CO_2_)] by X-ray diffraction. We tentatively formulate **5** as a Ce(IV) bis(phenylacetate) complex, [Ce(L_OEt_)_2_(PhCH_2_CO_2_)_2_].

**Figure 1:**
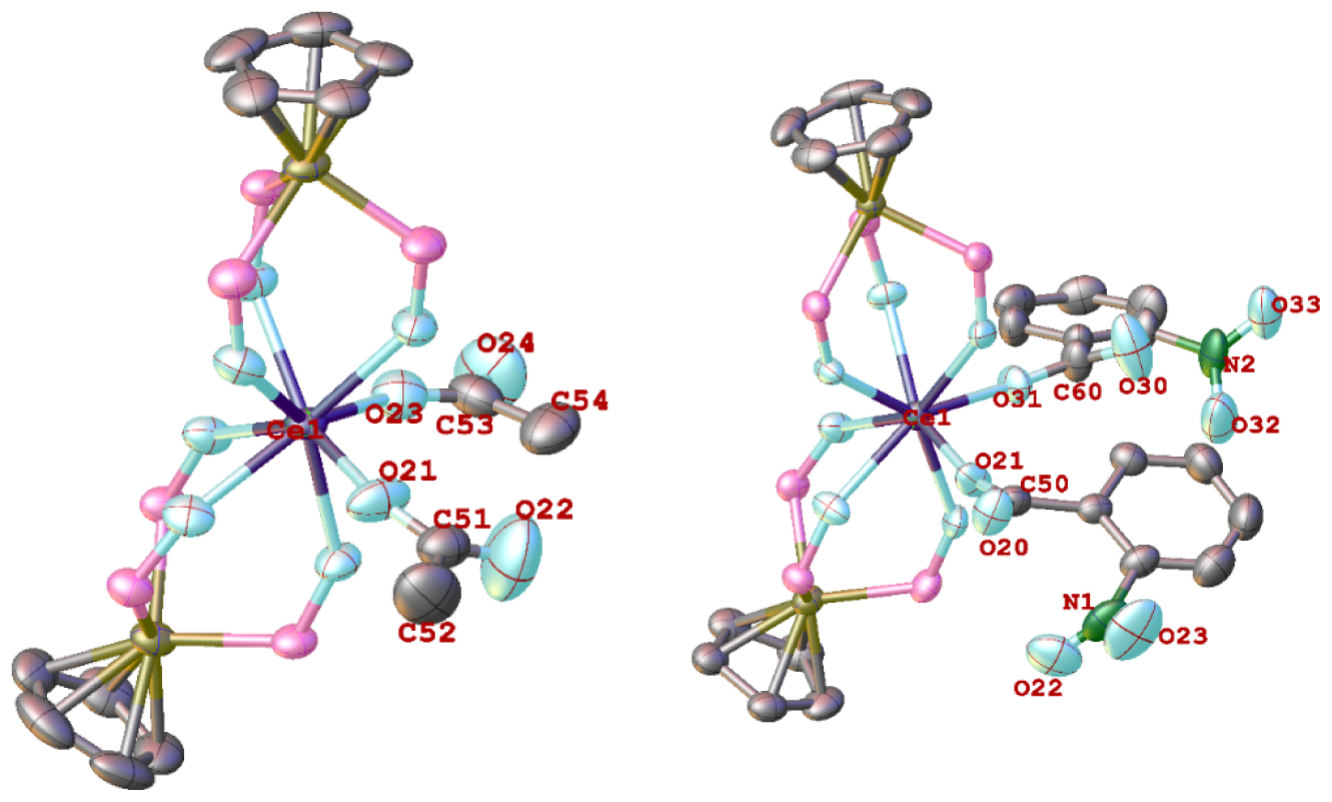
Molecular structures of the Ce(IV) carboxylate complexes **2** (left) and **4** (right). Hydrogen atoms and ethoxy groups of L_OEt_^–^ ligands are omitted for clarity. Ellipsoids are drawn at 40% probability level. Selected bond lengths (Å) and angles (deg): for **2**, Ce–O(L_OEt_) 2.346(4)-2.361(4), Ce1–O23 2.226(5), Ce1–O21 2.260(5); C53–O23–Ce1 152.0(5), C51–O21–Ce1 151.1(5). For **4**: Ce–O(L_OEt_) 2.298(4)-2.372(4), Ce1–O21 2.235(4), Ce1–O31 2.255(4); C50–O21–Ce1 164.9(5), C60–O31–Ce1 165.3(5).

The molecular structures of the Ce(IV) carboxylates **2** and **4** are shown in Figure 1. In both complexes, the Ce atom is coordinated with two tripod and two unidentate carboxylate ligands. No interactions were found between Ce and the ortho nitro substituents of the benzoate ligands in **4**. The Ce–O(carboxylate) distances in **2** [2.226(5) and 2.260(5) Å] and **4** [2.235(4) and 2.255(4) Å] are shorter than those for the κ^1^-pivalate ligands in the cluster [Ce_6_O_8_(^t^BuCO_2_)_8_(dien)_4_] [2.429(9) (Å), dien = diethylenetriamine].^26^

**Figure 2:**
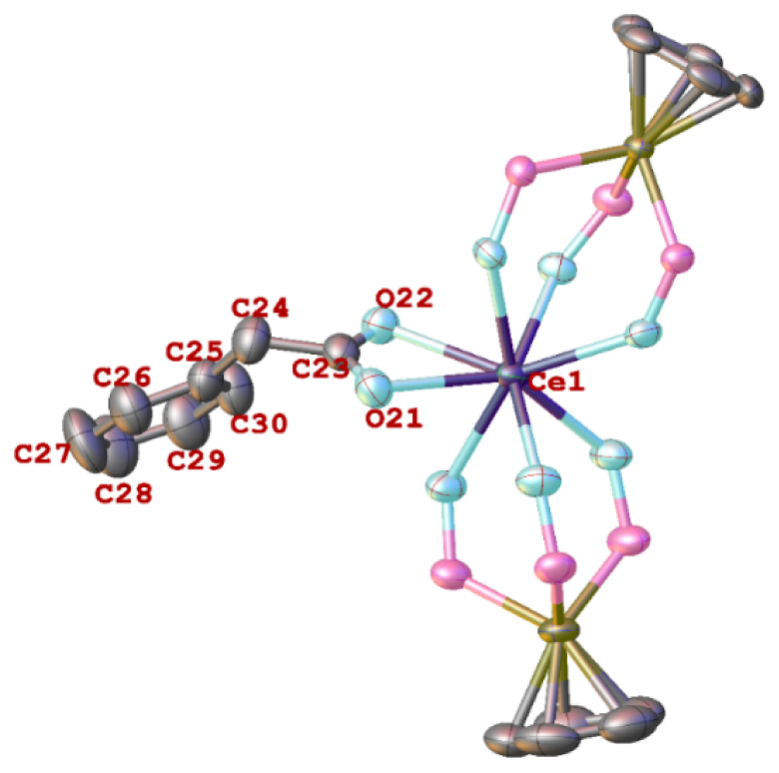
Molecular structure of [Ce (L_OEt_)_2_(OCOCH_2_Ph)] (**6**). Hydrogen atoms and ethoxy groups of L_OEt_^–^ ligands are omitted for clarity. Ellipsoids are drawn at 40% probability level. Selected bond lengths (Å) and angles (deg): Ce–O(L_OEt_) 2.409(3)-2.511(3), Ce1–O21 2.572(4), Ce1–O22 2.531(4); C23–O21–Ce1 92.0(3), C23–O22–Ce1 94.2(3).

The structure of the Ce(III) phenylacetate complex **6** is shown in Figure 2. Unlike **2** and **4**, the phenylacetate ligand in **6** binds to Ce in a κ^2^-*O*,*O*’ mode. The carboxylate Ce–O bond distances in **6** [2.531(4) and 2.572(4) Å] are longer than those in the Ce(IV) counterparts owing to the larger ionic radius of Ce^3+^ relative to Ce^4+^.

### Sulfamate and Amido Complexes

**Scheme 3:**
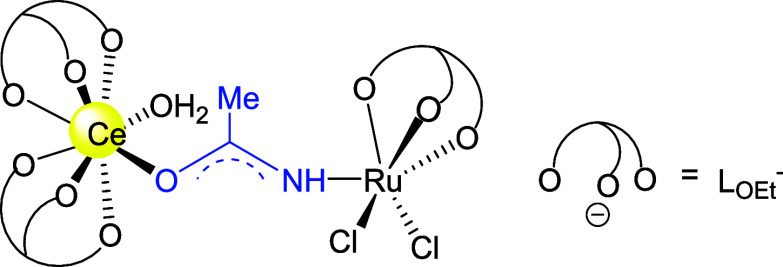
Structure of an Amidate-Bridged Ce(III)/Ru(III) Complex^27^

Attempts have been made to synthesize Ce(IV) amido complexes via protonolysis of **1** with compounds containing acidic NH_2_ groups. Treatment of **1** with sulfamic acid afforded [Ce(L_OEt_)_2_(SO_3_NH_2_)_2_] (**7**), which, to our knowledge, is the first molecular cerium sulfamate complex. No reaction was found between **1** and acetamide or methanesufonamide, but **1** reacted with more acidic trifluoroacetamide and trifluoromethanesulfonamide readily to yield [Ce(L_OEt_)_2_(CF_3_CONH)_2_] (**8**) and [Ce(L_OEt_)_2_(CF_3_SO_2_NH)_2_] (**9**), respectively. **7**–**9** exhibited sharp ^31^P NMR signals at δ 117–120 ppm that are indicative of the Ce(IV) formulation. It may be noted that in previous work, we have isolated an acetamide adduct of a Ce(IV) oxo complex, [Ce(L_OEt_)_2_(O)(H_2_O)]·MeCONH_2_,^17^ showing that acetamide cannot be deprotonated by the Ce = O group. On the other hand, the reaction of [Ce(L_OEt_)_2_(O)(H_2_O)]·MeCONH_2_ with a Ru(VI) nitride, [Ru(L_OEt_)(N)Cl_2_], resulted in elimination of NO and formation of a heterometallic Ce(III)/Ru(III) complex, [(L_OEt_)_2_(H_2_O)Ce{μ-*O*,*N*-MeC(O)NH}Ru(L_OEt_)Cl_2_], bearing a bridging amidate ligand (Scheme 3).^27^ Apparently, the binding to electrophilic Ru(III) facilitates the deprotonation of acetamide and the formation of the Ce/Ru amidate complex.

**Figure 3:**
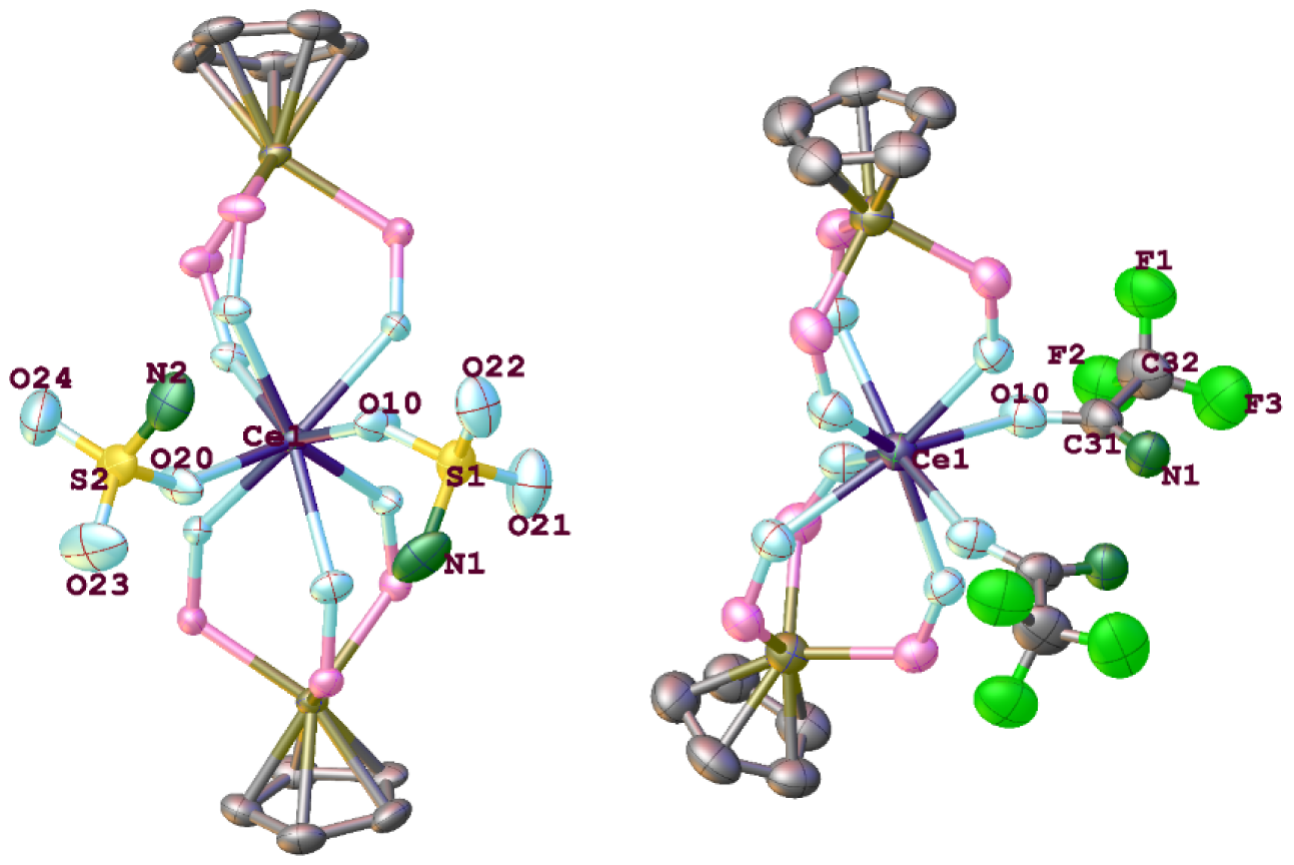
Molecular structures of **7** (left) and **8** (right). Hydrogen atoms and ethoxy groups of L_OEt_^–^ ligands are omitted for clarity. Ellipsoids are drawn at 40% probability level. Selected bond lengths (Å) and angles (deg): for **7**, Ce–O(L_OEt_) 2.273(3)-2.384(3), Ce1–O10 2.349(4), Ce1–O20 2.312(4); S1–O10–Ce1 141.0(3), S2–O20–Ce1 150.6(3). For **8**, Ce–O(L_OEt_) 2.446(4)-2.453(4), Ce1–O10 2.582(4), O10–C31 1.238(7), C31–N1 1.251(8); C31–O10–Ce1 138.9(4), O10–C31–N1 129.3(6).

The crystal structures of **7** and **8** are shown in Figure 3. The Ce atom in both complexes is 8-coordinated featuring two η^1^-bonded monoanionic sulfamate/amidate ligands. Although X-ray crystallography cannot unambiguously differentiate between an O from an N atom, it is reasonable to assume that the sulfamate/amidate ligands in **7** and **8** bind to Ce via an O instead of an N atom, considering the hardness of Ce^4+^. In **7**, the Ce–O(S) bond distances [2.349(4) and 2.312(4) Å] compare well with those in [Ce_2_(OH)_2_(H_2_O)_2_(SO_4_)_3_] (av. 2.328 Å).^28^ The bridged S–O(Ce) bonds [1.482(4) and 1.474(4) Å] are longer than the terminal S=O bonds [1.419(6) – 1.432(6) Å]. The S–N bond lengths [1.618(7) and 1.629(7) Å] are consistent with S–N single bonds. The Ce–O(amidate) distance in **8** [2.582(4) Å] is rather long possibly due to steric effects. The C–O [1.238(7) Å] and C–N [1.251(8) Å] distances of the amidate ligands in **8** are short, indicating π conjugation in the O–C–N unit, as shown in Scheme 2. A preliminary X-ray diffraction study revealed that **9** is a Ce(IV) complex containing two O-bonded sulfonamido ligands (Figure S6).

### Reactions of 1 with Aryl Alcohols

**Scheme 4:**
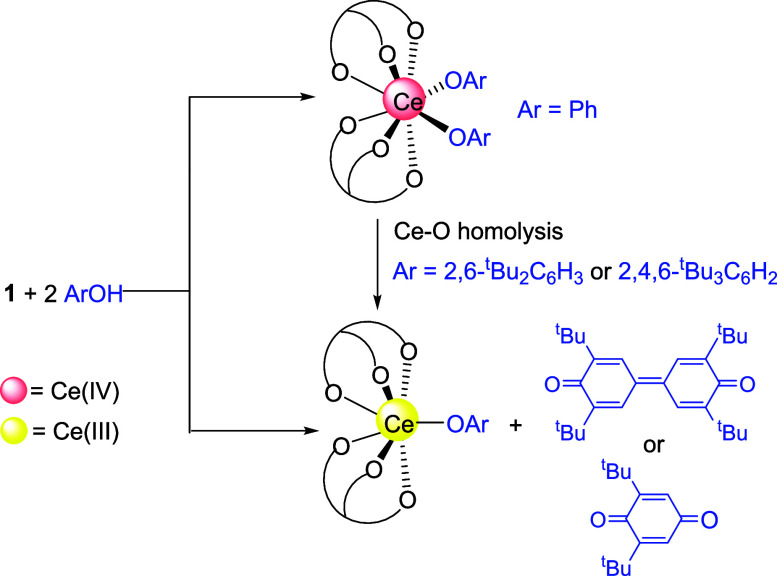
Reactions of **1** with Substituted Phenols

**Scheme 5:**
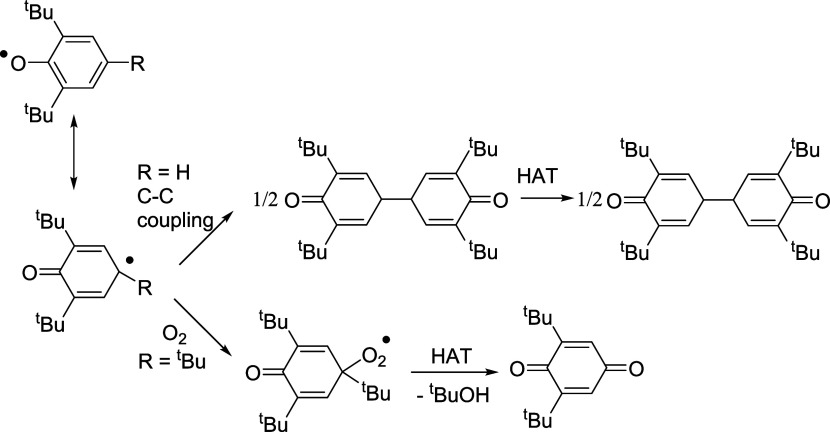
Proposed Pathways for Formation of the Quinone Products in Scheme 4

Protonolysis of **1** with nonbulky aryl alcohols, such as phenol, gave Ce(IV) bis(aryloxide) complexes [Ce(L_OEt_)_2_(OAr)_2_] (e.g., Ar = phenyl) (Scheme 4).^16^ Like Ce(IV)-L_OEt_ alkoxide complexes,^15^**1** is capable of oxidizing 2,6-disubstituted phenols. For example, treatment of **1** with 2,6-di-*tert*-butylphenol afforded the previously reported Ce(III) aryloxide complex [Ce(L_OEt_)_2_(OC_6_H_3_^t^Bu_2_-2,6)]^15^ along with 2,6-di-*tert*-butylbiquinone. Similarly, the reaction of **1** with 2,4,6-tri-*tert*-butylphenol gave [Ce(L_OEt_)_2_(OC_6_H_2_^t^Bu_3_-2,4,6)] and 2,6-di-*tert*-butyl-1,4-benzoquinone (Scheme 4). As proposed in our previous work,^15,16^ the oxidation of the 2,6-disubstituted phenols by **1** involved the formation of unstable Ce(IV) bis(aryloxide) intermediates, which due to steric effects, underwent Ce–O homolysis to give Ce(III) and aryloxy radicals. Then the generated aryloxy radical rearranged to a C-centered radical that either (a) underwent C–C coupling (for 2,6-di-*tert*-butylphenol) or (b) was oxidized by adventitious oxygen (for 2,4,6-tri-*tert*-butylphenol) to yield the quinone product and *tert*-butanol (Scheme 5). Sterically induced Ce–O homolysis of Ce(IV) 2,6-disubstituted aryloxide complexes has been demonstrated by us previously.^16^

### C–H Oxidation by 1

#### 9,10-Dihydroanthracene

**Scheme 6:**
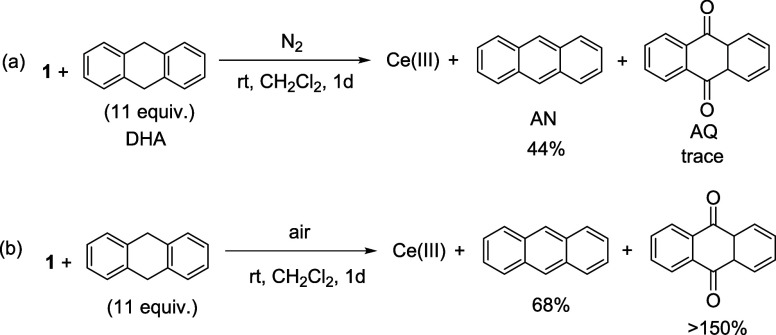
Oxidation of DHA by **1** (Yield Relative to **1**)

Treatment of **1** with DHA in CH_2_Cl_2_ under nitrogen led to formation of a Ce(III) species and anthracene (AN) in 44% yield (with respect to **1** that is assumed to be a 1-electron oxidant) (Scheme 6a). The Ce(III) product that exhibited a ^31^P NMR signal at δ^P^ 154 ppm is tentatively formulated as a Ce(III) bicarbonate, [Ce(L_OEt_)_2_(HCO_3_)].^25^ In addition, a small amount of anthraquinone (AQ) that was formed by oxidation of anthracene radical with adventitious oxygen was detected. When the reaction was carried out under air, AQ was produced in over 150% yield (Scheme 6b), showing that **1** can initiate the autoxidation of DHA via H-atom abstraction. No HAT was found between **1** and hydrocarbons that possess strong C–H bonds, such as cyclohexane (BDE = 99.5 kJ/mol).^29^ The oxidation of 2,6-di-*tert*-butylphenol by **1** (<30 min) was much faster than that of DHA (1 d) because the former involved proton transfer and subsequent coordination of the aryloxide ligands to Ce(IV) that facilitates the oxidation.

#### Tetrahydrofuran

While **1** is air-stable in common organic solvents such as acetonitrile and benzene, it decomposed readily in tetrahydrofuran (THF). Dissolution of **1** in THF in air resulted in a rapid color change from orange to yellow. ^31^P NMR spectroscopy indicated that **1** was completely reduced to a Ce(III) species (δ^P^ = 154 ppm), which is presumably the same Ce(III) product obtained from the HAT of **1** with DHA. By contrast, the reduction of **1** in THF under nitrogen was much slower. In a N_2_-filled glovebox at room temperature, only about 25% of **1** was reduced to Ce(III) in THF in 1 d (Figure S15, Supporting Information). The effects of oxygen and water on the stability of **1** in THF have been examined. No significant change was found when water was added to a THF solution of **1** under nitrogen while **1** was completely reduced to Ce(III) after bubbling the solution with oxygen for 1 min (Figure S16), suggesting that oxygen instead of water is responsible for the rapid reduction of **1** in THF in air.

When **1** was dissolved in THF in air, a yellow solution containing a Ce(III) species was formed. Mass spectrometry indicated that the yellow solution contained tetrahydrofuran hydroperoxide (*m*/*z* 103.04), which was also detected using a peroxide test strip (see Supporting Information). No peroxide species was detected in a THF solution of **1** under nitrogen. Furthermore, we found that treatment of **1** with tetrahydrofuran hydroperoxide^30^ in benzene under nitrogen resulted in immediate reduction of Ce(IV) to Ce(III) with concomitant evolution of a gas, presumably oxygen. The reaction of tetrahydrofuran hydroperoxide with **1** probably proceeded by a similar pathway as the oxidation of arylalcohols with **1**, involving an HAT step, followed by oxygen elimination. Therefore, we hypothesize that tetrahydrofuran hydroperoxide is the actual reducing agent responsible for the rapid reduction of **1** in THF in air.

**Scheme 7:**
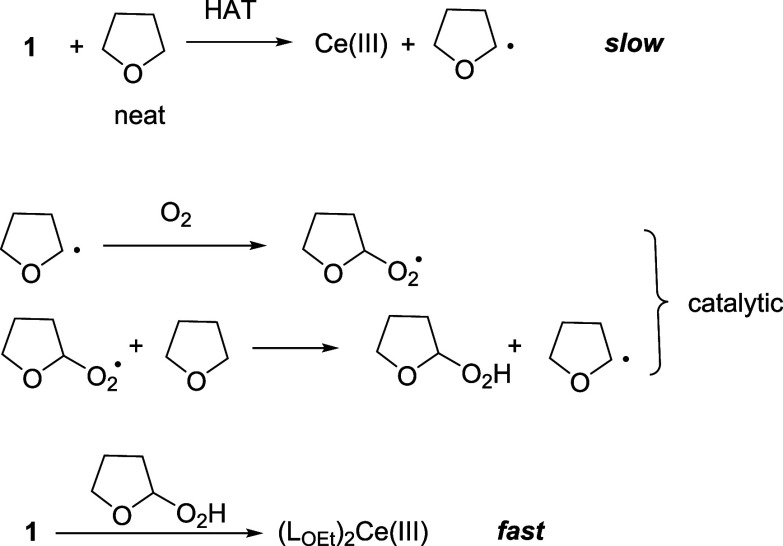
Proposed Mechanism for the Reduction of **1** in THF in Air

Taken together, a mechanism is proposed for the reduction of **1** in THF in air (Scheme 7). The first step is the H atom abstraction of THF by **1**, which is expected to be slow given the high C–H BDE of THF (92 kJ/mol).^29^ In the presence of air/oxygen, the generated tetrahydrofuran radical reacts with oxygen to give tetrahydrofuran hydroperoxide catalytically, which reduces **1** to Ce(III) rapidly. Preliminary studies showed that **1** was also reduced to Ce(III) in ether solvents such as diethyl ether and 1,2-dimethoxyethane in air rapidly, presumably via the reduction of the Ce(IV) carbonate with hydroperoxide species.

### Photoreduction of Cerium(IV) Carboxylates

Ce(IV)-L_OEt_ carboxylate complexes underwent photoreduction when irradiated with blue LED light. For example, irradiation of **2** in C_6_D_6_ with blue LED light for 30 min resulted in formation of a Ce(III) species, presumably [Ce(L_OEt_)_2_(CH_3_CO_2_)], in ca. 75% yield according to ^31^P NMR spectroscopy. By contrast, the Ce(IV) 2-nitrobenzoate complex **4** is photostable; no change was found in the ^31^P NMR spectrum after **4** in C_6_D_6_ was irradiated with blue LED light for 24 h. The photostability of **4** can be rationalized in terms of stabilization of the HOMO of the benzoate ligand by the electron-withdrawing nitro group, which inhibits the LMCT-induced Ce–O homolysis. Irradiation of a 3:1 mixture of **5** and **6** (vide supra) in benzene with blue LED light in air for 30 min led to complete reduction of **5** to **6** along with formation of benzaldehyde that was identified by GC-MS, indicating that the photoreduction involved Ce–O homolysis and decarboxylation of the carboxylate radical.

### Ce-Catalyzed Photodecarboxylative Oxygenation of Arylacetic Acids

**Scheme 8:**
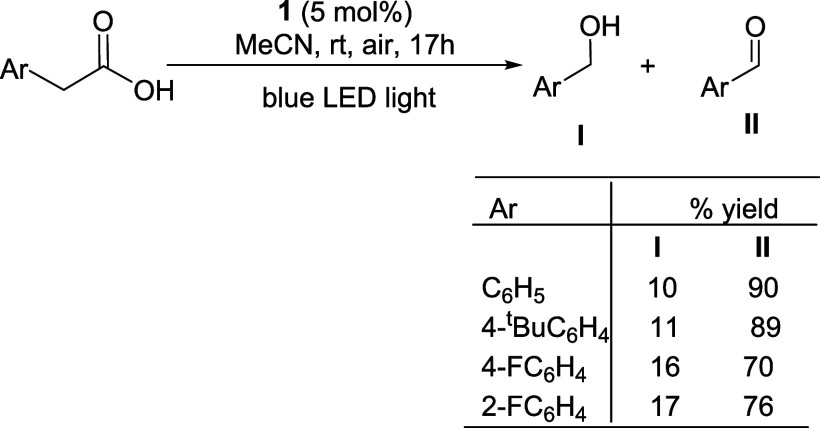
Ce-catalyzed Decarboxylative Oxygenation of Arylacetic Acids Reaction conditions: **1** (3.1 μmol), acrylacetic acid (0.060 mmol), MeCN (3 mL), air, room temperature, blue LED (5 W). Yields relative to arylacetic acid were determined by GLC.

Since Ce(IV) carboxylates can be easily accessed from **1** and carboxylic acids, air-stable **1** has been used as a catalyst precursor for decarboxylative oxygenation of carboxylic acids (Scheme 8). For example, photolysis of phenylacetic acid with blue LED light in the presence of 5 mol % of **1** under air afforded a 1:9 mixture of benzyl alcohol and benzaldehyde quantitatively. The substituent effect on the Ce-catalyzed photodecarboxylative oxygenation has been studied. While the fluorine substituent (both ortho and para) in the arylacetic acids slightly increased the relative yield of the alcohol products, the para *tert*-butyl group seemed to have no effect on the selectivity of the decarboxylative oxygenation.

**Scheme 9:**
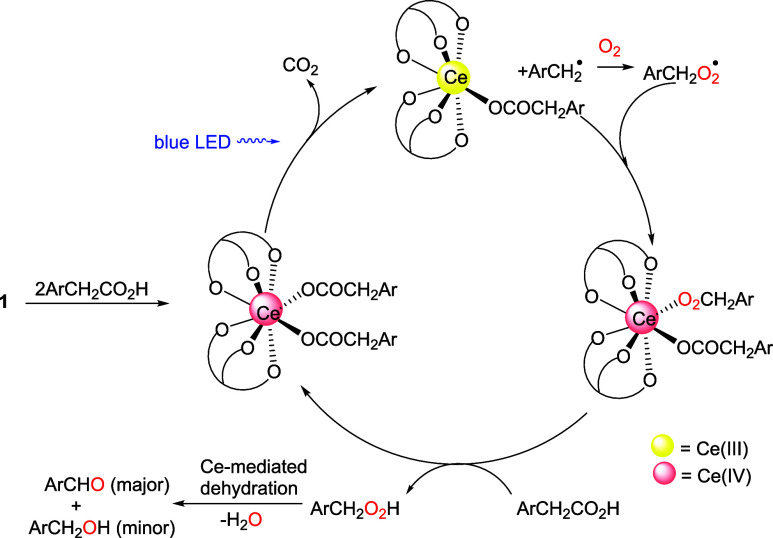
Proposed Mechanism for **1**-Catalyzed Decarboxylative Oxygenation of Arylacetic Acids

Previous studies have indicated that Ce-mediated decarboxylation of carboxylic acids involved LMCT-induced Ce–O homolysis of Ce(IV) carboxylate complexes.^19,20,21^ In addition, alkyl hydroperoxides have been shown to be key intermediates in the decarboxylative oxygenation of carboxylic acids to alcohols and alehydes.^21d^ Therefore, a catalytic cycle is proposed for the **1**-catalyzed photodecarboxylative oxygenation of arylacetic acids (Scheme 9). Protonolysis of **1** with ArCH_2_CO_2_H afforded a Ce(IV) bis(arylacetate) complex that upon irradiation with blue LED underwent Ce–O homolysis and decarboxylation. Reaction of the generated ArCH_2_^•^ radical with oxygen gave an ArCH_2_O_2_^•^ radical that reoxidized Ce(III) to a Ce(IV)–OOCH_2_Ar species. Protonolysis of Ce(IV)–OOCH_2_Ar with ArCH_2_CO_2_H gave a hydroperoxide, ArCH_2_O_2_H, which, in the presence of the cerium complex, was dehydrated to ArCHO along with a minor product, ArCH_2_OH. Our result is consistent with previous work on cerium-mediated dehydration of alkyl hydroperoxide species, in which aldehydes were obtained as major products.^18,20a,21d^

## Conclusions

We have found that the Ce(IV) carbonate complex **1** is a good starting material for the synthesis of Ce(IV)-L_OEt_ complexes containing weakly basic O-donor ligands, such as carboxylate, sulfamate, and amidate. Unlike the acetate analogue, the Ce(IV) phenylacetate **5** undergoes facile decarboxylation in the dark at room temperature via Ce–O homolysis. Owing to the basicity of the carbonate ligand and oxidizing power of Ce(IV), **1** can serve as a PCET oxidant for O–H (e.g., phenols) and C–H (e.g., DHA) oxidations. While **1** is air-stable in common organic solvents, it is readily reduced in THF and ether solvents via HAT. In air, **1** reacted with THF to give THF hydroperoxide that reduces **1** to Ce(III) rapidly. Upon irradiation with blue LED light, the Ce(IV)-L_OEt_ carboxylate complexes undergo facile LMCT-induced decarboxylation. **1** proved be a convenient, air-stable catalyst precursor for photodecarboxylative oxygenation of aryl acetic acids. The results of this work demonstrate that with judicious control of the basicity and donor strength of ligands, oxidizing tetravalent cerium compounds can serve as HAT reagents that can find applications in organic transformations.

## Experimental Section

### General Considerations

All manipulations were performed under dinitrogen using standard Schlenk techniques. Solvents were distilled from Na/benzophenone (hexane, tetrahydrofuran, and Et_2_O) or CaH_2_ (CH_2_Cl_2_ and MeCN) prior to use. Substituted phenols were purchased from commercial sources and used as received. The compound [Ce(L_OEt_)_2_Cl_2_] was synthesized as described elsewhere.^17a^ Ag_2_CO_3_ was freshly prepared from AgNO_3_ and Na_2_CO_3_ in water. NMR spectra were recorded on a Bruker ARX 400 spectrometer operating at 400, 376.5, and 162 MHz for ^1^H, ^19^F, and ^31^P, respectively. Chemical shifts (δ, ppm) were reported with reference to SiMe_4_ (^1^ H), CF_3_C_6_H_5_ (^19^F), and H_3_PO_4_ (^31^P). IR spectra (KBr) were recorded on a PerkinElmer 16 PC FT-IR spectrophotometer. Mass spectrometry was performed on a Waters Xevo G2 Q-Tof mass spectrometer equipped with a standard ESI interface. Gas chromatography was performed on a Bruker 430-GC instrument equipped with an FID detector. Photolysis of Ce(IV) carboxylate complexes was conducted using blue LED lights of a Aldrich Micro Photochemical Reactor (λ = 435–445 nm, 5–6 W). Elemental analyses were performed by Medac Ltd., Surrey, UK.

### Synthesis of [Ce(L_OEt_)_2_(CO_3_)] (1)

A mixture of [Ce(L_OEt_)_2_Cl_2_] (50.0 mg, 0.039 mmol) and Ag_2_CO_3_ (10.8 mg, 0.039 mmol) in MeCN (10 mL) was stirred at room temperature for 30 min. The resulting solution was filtered, and the solvent was removed in vacuo to give an orange solid. Recrystallization from CH_2_Cl_2_/hexane gave orange crystals that were identified as the previously reported carbonate compound [Ce(L_OEt_)_2_(CO_3_)]^17^ by NMR spectroscopy and X-ray diffraction. Yield: 42.6 mg (86%). ^1^H NMR (400 MHz, C_6_D_6_, 25 °C): δ 1.26 (t, *J* = 8.0 Hz, 36H, CH_3_), 4.30 (m, 24H, CH_2_), 4.94 (s, 10H, Cp). ^31^P{^1^H} NMR (162 MHz, C_6_D_6_, 25 °C): δ 116.62 (s).

### Reaction of 1 with Acetic Acid

A mixture of **1** (30.0 mg, 0.024 mmol) and acetic acid (2.7 μL, 0.047 mmol) in MeCN (10 mL) was stirred at room temperature for 30 min. The solvent was removed in vacuo to give a yellow solid that was extracted with hexane. Recrystallization from hexane gave orange crystals that were characterized as the previously reported Ce(IV) acetate compound [Ce(L_OEt_)_2_(CH_3_CO_2_)_2_] (**2**) by NMR spectroscopy. Yield: 24.3 mg (78%). Alternatively, **2** can be synthesized by salt metathesis of [Ce(L_OEt_)_2_Cl_2_] with Ag(CH_3_CO_2_) in MeCN and recrystallized from hexane. ^1^H NMR (400 MHz, C_6_D_6_, 25 °C): δ 1.302–1.338 (t, *J* = 8.0 Hz, 36H, CH_3_ of L_OEt_), 2.37 (s, 6H, CH_3_ of acetate), 4.35–4.41 (m, 24H, CH_2_), 5.01 (s, 10H, Cp). ^31^P{^1^H} NMR (162 MHz, C_6_D_6_, 25 °C): δ 115.978 (s). Anal. Calcd for C_38_H_76_CeCo_2_O_22_P_6_: C, 34.35; H, 5.76. Found: C, 33.67; H, 5.68.

### Synthesis of [Ce(L_OEt_)_2_(PhCO_2_)_2_] (3)

A mixture of **1** (30.0 mg, 0.024 mmol) and benzoic acid (5.8 mg, 0.047 mmol) in MeCN (10 mL) was stirred at room temperature for 30 min. Evaporation of the solvent and recrystallization from hexane at −20 °C afforded an orange solid. Yield: 26.1 mg (77%). ^1^H NMR (400 MHz, C_6_D_6_, 25 °C): δ 1.22–1.25 (t, *J* = 8.0 Hz, 36H, CH_3_ of L_OEt_), 4.33–4.37 (m, 24H, CH_2_), 4.98 (s, 10H, Cp), 7.27 (br, 2H, H_p_), 7.40 (br, 4H, H_m_), 8.88 (br, 4H, H_o_). ^31^P{^1^H} NMR (162 MHz, C_6_D_6_, 25 °C): δ 116.28 (s). Anal. Calcd for C_48_H_80_CeCo_2_O_22_P_6_: C, 39.68; H, 5.55. Found: C, 39.34; H, 5.58.

### Synthesis of [Ce(L_OEt_)_2_(2-NO_2_C_6_H_4_CO_2_)_2_] (4)

A mixture of **1** (30.0 mg, 0.024 mmol) and 2-nitrobenzoic acid (7.9 mg, 0.047 mmol) in MeCN (10 mL) was stirred at room temperature for 30 min. The solvent was evaporated to dryness to give an orange solid. Recrystallization from CH_2_Cl_2_/hexane afforded orange crystals. Yield: 27.3 mg (75%). ^1^H NMR (400 MHz, CD_3_CN, 25 °C): δ 1.16–1.20 (t, *J* = 8.0 Hz, 36H, CH_3_), 4.05 (m, 24H, CH_2_), 5.10 (s, 10H, Cp), 7.51–7.59 and 7.95 (br, 8H, Ar). ^31^P{^1^H} NMR (162 MHz, CD_3_CN, 25 °C): δ 116.98–120.11 (br.). Anal. Calcd for C_48_H_78_CeCo_2_N_2_O_26_P_6_: C, 37.36; H, 5.10; N, 1.82. Found: C, 37.20; H, 5.14; N, 1.78.

### Reaction of 1 with Phenylacetic Acid

A mixture of **1** (30.0 mg, 0.024 mmol) and phenylacetic acid (6.5 mg, 0.048 mmol) in MeCN (10 mL) was stirred at room temperature for 30 min. Evaporation of the solvent and extraction with hexane afforded an orange solid, which according to ^31^P NMR spectroscopy, consisted of a 3:1 mixture of a Ce(IV) (**5**) and a Ce(III) (**6**) species. The **5**:**6** ratio of the mixture in benzene-*d*_6_ dropped from 3:1 to 1:2 in 3 h. GC-MS analysis revealed that the resulting mixture contained benzaldehyde. Recrystallization from hexane at −20 °C for 3 d led to isolation of yellow crystals of **6** that were identified as [Ce^III^(L_OEt_)_2_(PhCH_2_CO_2_)]. Yield: 22.5 mg (71%). For **6**, ^1^H NMR (400 MHz, CDCl_3_, 25 °C): δ −0.04 (br, 36H, CH_3_), 1.39 (br, 2H, CH_2_), 1.88–2.59 (br, 24H, CH_2_), 5.36 (br, 2H, H_o_), 6.47 (br, 2H, H_m_), 6.70 (br, 1H, H_p_), 10.57 (br, 10H, Cp). ^31^P{^1^H} NMR (162 MHz, CDCl_3_, 25 °C): δ 153.84 (s). Anal. Calcd for C_42_H_77_CeCo_2_O_20_P_6_: C, 37.48; H, 5.77. Found: C, 38.10; H, 5.82.

### Reaction of a of Mixture 5:6 with TEMPO

To a ca. 3:1 mixture of **5**:**6** in benzene prepared as described earlier was added (2,2,6,6-tetramethylpiperidin-1-yl)oxyl (TEMPO) (2 equiv. with respect to **1**), and the resulting solution was stirred in the dark at room temperature under nitrogen for 1 h. LC-MS analysis indicated that TEMPO-benzyl was produced (*m*/*z*: [*M* + H]^+^ calcd for C_16_H_26_NO: 248.2009; found: 248.2008).
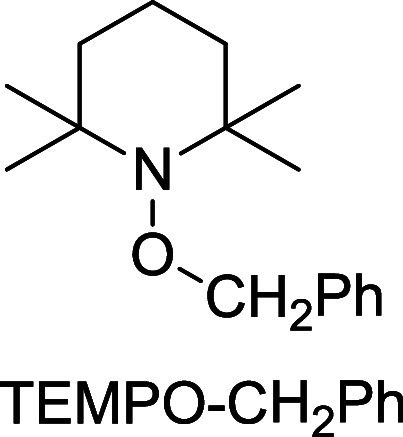


### Synthesis of [Ce(L_OEt_)_2_(SO_3_NH_2_)_2_] (7)

A mixture of **1** (30.0 mg, 0.024 mmol) and sulfamic acid (4.6 mg, 0.047 mmol) in MeCN (10 mL) was stirred at room temperature for 2 h. The solvent was pumped off, and the residue was washed with hexane and Et_2_O and extracted with CH_2_Cl_2_ (ca. 10 mL). Recrystallization from CH_2_Cl_2_/Et_2_O/hexane at room temperature afforded red crystals. Yield: 26.9 mg (81%). ^1^H NMR (400 MHz, CD_3_CN, 25 °C): δ 1.24–1.28 (t, *J* = 8.0 Hz, 36H, CH_3_), 4.10–4.15 (m, 24H, CH_2_), 4.61 (br, 4H, NH_2_), 5.16 (s, 10H, Cp). ^31^P{^1^H} NMR (162 MHz, CD_3_CN, 25 °C): δ 120.21 (s). Anal. Calcd for C_34_H_74_CeCo_2_N_2_O_24_P_6_S_2_: C, 29.11; H, 5.32; N, 2.00. Found: C, 28.82; H, 5.16; N, 2.03.

### Synthesis of [Ce(L_OEt_)_2_(CF_3_CONH)_2_] (8)

A mixture of **1** (30.0 mg, 0.024 mmol) and trifluoroacetamide (5.4 mg, 0.048 mmol) in MeCN (10 mL) was stirred at room temperature for 2 h. The solvent was pumped off, and the residue was extracted with hexane. Concentrating the filtrate and cooling at −20 °C afforded orange crystals. Yield: 21.1 mg (62%). ^1^H NMR (400 MHz, C_6_D_6_, 25 °C): δ 1.25–1.28 (overlapping t, 36H, CH_3_), 4.35 (m, 24H, CH_2_), 4.90 (s, 10H, Cp). ^19^F{^1^H} NMR (376 MHz, C_6_D_6_, 25 °C): δ −75.99 (s). ^31^P{^1^H} NMR (162 MHz, C_6_D_6_, 25 °C): δ 117.36 (s). Anal. Calcd for C_38_H_72_CeCo_2_F_6_N_2_O_20_P_6_·2H_2_O C, 31.03; H, 5.21; N, 1.90. Found: C, 30.11; H, 5.21; N, 1.31. Despite two attempts, we did not obtain good analytical data for the compound. Nevertheless, the identity of the Ce(IV) acetamido compound has been confirmed by NMR spectroscopy and X-ray diffraction.

### Synthesis of [Ce(L_OEt_)_2_(CF_3_SO_2_NH)_2_] (9)

This compound was prepared similarly as for **8** using trifluoromethanesulfonamide in place of trifluoroacetamide. Recrystallization from hexane −20 °C afforded red crystals. Yield: 23.7 mg (67%). ^1^H NMR (400 MHz, CDCl_3_, 25 °C): δ 1.25–1.29 (overlapping t, 36H, CH_3_), 4.28 (br, 24H, CH_2_), 4.89 (s, 10H, Cp). ^19^F{^1^H} NMR (376 MHz, CDCl_3_, 25 °C): δ −79.58 (s). ^31^P{^1^H} NMR (162 MHz, C_6_D_6_, 25 °C): δ 117.69 (s). Anal. Calcd for C_36_H_72_CeCo_2_F_6_N_2_O_22_P_6_S_2_: C, 28.69; H, 4.82; N, 1.86. Found: C, 27.56; H, 4.69; N, 2.06. Despite two attempts, we were not able to obtain good analytical data for the compound. Nevertheless, the identity of the Ce(IV) sulfonamido compound has been confirmed by NMR spectroscopy and X-ray diffraction.

### Reaction of 1 with Phenol

A mixture of **1** (30.0 mg, 0.024 mmol) and phenol (4.6 mg, 0.049 mmol) in MeCN (10 mL) was stirred at room temperature for 30 min. The solvent was evaporated to dryness to give a purple solid, and the residue was extracted with hexane. Recrystallization from hexane gave purple crystals that were identified as the previously reported compound [Ce(L_OEt_)_2_(OPh)_2_]^16^ by NMR spectroscopy. Yield: 28.7 mg (87%). 1H NMR (400 MHz, CDCl_3_, 25 °C): δ 1.12 (t, *J* = 8.0 Hz, 36H, CH_3_), 3.89–4.02 (br, 24H, CH_2_), 4.97 (s, 10H, Cp), 6.30 (t, *J* = 8.0 Hz, 2H, H_p_), 6.63 (d, *J* = 8.0 Hz, 4H, H_o_), 7.02 (t, *J* = 8.0 Hz, 4H, H_m_). ^31^P{^1^H} NMR (162 MHz, CDCl_3_, 25 °C): δ 114.29 (s).

### Reaction of 1 with 2,6-Di-*tert*-Butylphenol

A mixture of **1** (30.0 mg, 0.024 mmol) and 2,6-di-*tert*-butylphenol (9.8 mg, 0.047 mmol) in MeCN (10 mL) was stirred at room temperature for 30 min. The orange color rapidly faded to yellow with the formation of a red precipitate. The solvent was pumped off, and the residue was extracted with hexane (5 mL). Concentrating the filtrate and cooling at −20 °C afforded yellow crystals that were characterized as the previously reported Ce(III) aryloxide [Ce^III^(L_OEt_)_2_(OC_6_H_3_*^t^*Bu_2_-2,6)]^15^ by NMR spectroscopy. The red precipitate has been identified as 3,3′,5,5′-tetra-*tert*-butyldiphenoquinone by NMR spectroscopy and GC-MS analysis (*m*/*z* 408.3, *M*^*+*^).
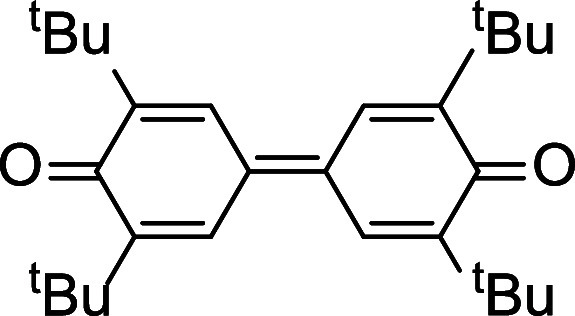


### Reaction of 1 with 2,4,6-Tri-*tert*-Butylphenol

A mixture of **1** (10.0 mg, 7.9 μmol) and 2,4,6-tri-*tert*-butylphenol (4.2 mg, 0.016 mmol) in MeCN (10 mL) was stirred at room temperature for 30 min. The orange color of the solution changed to pale yellow rapidly in 30 min. Concentrating the filtrate and cooling at −20 °C afforded yellow crystals that were characterized as the previously reported Ce(III) aryloxide [Ce(L_OEt_)_2_(OC_6_H_2_^t^Bu_3_-2,4,6)]^15^ by NMR spectroscopy. GC-MS analysis revealed that the reaction mixture contained 2,6-di-*tert*-butyl-1,4-benzoquinone (*m*/*z* 220.1, *M*^*+*^).
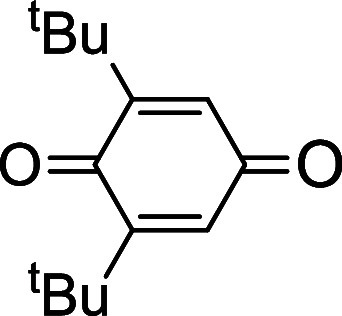


### Reaction of 1 with 9,10-Dihydroanthracene

A mixture of **1** (4.1 mg, 3.2 μmol) and DHA (6.3 mg, 0.035 mmol, 11 equiv) was stirred in CH_2_Cl_2_ (3 mL) at room temperature for 24 h, during which the color of the solution changed from orange to yellow. The yield of the major organic product, anthracene, was determined to be 44% (relative to Ce, assuming that **1** is a 1-electron oxidant) by GLC using 1,3,5-trimethoxybenzene as internal standard. In addition, a trace amount of anthraquinone was detected. When the reaction of **1** with DHA was conducted in air, a ca. 1:2 mixture of anthracene and anthraquinone was formed in over 220% yield.

### Reduction of 1 in Tetrahydrofuran Under Nitrogen

To **1** (10 mg, 7.9 μmol) in a J-Young NMR tube was added distilled THF (1 mL) in a nitrogen-filled glovebox. After 24 h, ^31^P NMR spectroscopy showed that about 25% of **1** (δ 115.1 ppm) was reduced to a Ce(III) species, [Ce^III^(L_OEt_)_2_(HCO_3_)], (δ 151.52 ppm).

### Reduction of 1 in THF in Air

To **1** (10 mg, 7.9 μmol) was added in 5 mL of distilled THF in air. The color of the solution immediately changed from orange to yellow. ^31^P NMR spectroscopy indicated that **1** has been completely converted to a Ce(III) species (δ 151.52 ppm). MS-MS analysis revealed that the yellow solution contained tetrahydrofuran hydroperoxide (C_4_H_8_O_3_, *m*/*z* = 103.0385, *M*^+^) (Figure S17), which was also detected by a Quantofix peroxides test stick. No hydroperoxide was found in a THF solution of **1** under nitrogen.

### Effect of Oxygen on the Reduction of 1 in Tetrahydrofuran

To **1** (5 mg) in a 5 mm NMR tube was added in 1 mL of distilled THF in a glovebox. The NMR tube sealed with a septum was taken out from the glovebox, and oxygen was bubbled into the solution through a syringe needle for 1 min. ^31^P NMR spectroscopy indicated that **1** was reduced to Ce(III) species (Figure S16).

### Effect of Water on the Reduction of 1 in Tetrahydrofuran

A solution of **1** in THF in a 5 mm NMR tube was prepared as described earlier and the ^31^P{^1^H} NMR spectrum was recorded. To the solution was added 1 drop of water using a syringe. No significant change was found in the ^31^P NMR spectrum.

### Reaction of Tetrahydrofuran Hydroperoxide with 1

To **1** (5 mg) in C_6_D_6_ (1 mL) in a 5 mm NMR tube was added 1 equiv of tetrahydrofuran hydroperoxide^30^ under nitrogen. Gas bubbles, presumably oxygen, were formed immediately, as the color of the solution changed from orange to yellow. ^31^P NMR spectroscopy indicated that **1** has been completely reduced to Ce(III).

### Photolysis of 2

A solution of **2** (4.7 mg, 3.5 μmol) in degassed C_6_D_6_ (1 mL) in a 5 mm NMR tube was irradiated with blue LED light for 30 min, during which the orange solution turned pale yellow. ^31^P NMR spectroscopy indicated the pale yellow solution contained a 1:3 mixture of **2** and a Ce(III) species (δ 153.59 ppm), presumably the Ce(III) acetate [Ce(L_OEt_)_2_(CH_3_CO_2_)].

### Photolysis of Ce(IV) Phenylacetate in Air

A ca. 3:1 mixture of **5** and **6** (prepared in situ from 5 mg of **1** and 2 equiv. phenylacetic acid in 1 mL of benzene-*d*_6_) in a 5 mm NMR tube was irradiated with blue LED light under air for 30 min. The ^31^P NMR spectrum of the final yellow solution displayed the resonance of **6** only. GC-MS analysis indicated benzaldehyde was produced.

### Ce-Catalyzed Photodecarboxylative Oxygenation of Arylacetic Acids

To **1** (4.0 mg, 3.1 μmol) in in MeCN (3 mL) in a 20 mL vial was added arylacetic acid (0.063 mmol) in air. The mixture was irradiated with blue LED light and stirred at room temperature for 17 h. The yields of the aldehyde and alcohol products were determined by GLC using 1,3,5-trimethoxybenzene as internal standard.

### X-ray Crystallography

Crystallographic data and experimental details for complexes **2**, **4** and **6**-**8** and are summarized in Tables S1 (Supporting Information). Single crystals of **2**, **6** and **8** were grown from concentrated hexane solutions at −20 °C, whereas those of **4** and **7** were grown from CH_2_Cl_2_/hexane and CH_2_Cl_2_/Et_2_O/hexane, respectively, at room temperature. A suitable crystal was selected and mounted on a SuperNova, Cu–K_∝_, with Atlas diffractometer. The crystals were kept at 173.00(10) K during data collection. Using Olex2,^31^ the structures were solved with the ShelXT^32^ structure solution program using Intrinsic Phasing and refined with the ShelXL^33^ refinement package using least-squares minimization. Atomic positions of non-hydrogen atoms were refined with anisotropic parameters and with suitable restraints. Disordered atoms were refined isotropically. Hydrogen atoms were generated geometrically and allowed to ride on their respective parent carbon atoms before the final cycle of least-squares refinement. Details of disorder of the crystal structures are given in the Supporting Information.

## Supporting Information Available

Additional crystallographic data, NMR, IR, and mass spectra (PDF)
