# Supplementary material for: Redox and Photochemical Reactivity of Cerium(IV) Carbonate and Carboxylate Complexes Supported by a Tripodal Oxygen Ligand
Source: Inorg Chem. 2025 Jan 15;64(3):1433–42. doi: 10.1021/acs.inorgchem.4c04602 (PMC11823459; doi:10.1021/acs.inorgchem.4c04602)
Supplement: Supplementary file 1 — ic4c04602_si_001.pdf [file ic4c04602_si_001.pdf]

Supporting Information for

Redox and Photochemical Reactivity of Cerium(IV) Carbonate and Carboxylate Complexes Supported by a Tripodal Oxygen Ligand

Hoang-Long Pham, Xinxin Jiang, Qiaolin Yan, Yat-Ming So, Wan Chan, Herman H. Y. Sung, Ian D. Williams*, and Wa-Hung Leung*

Department of Chemistry, The Hong Kong University of Science and Technology, Clear Water Bay, Kowloon, Hong Kong, China

*Corresponding authors. E-mail: chwill@ust.hk, chleung@ust.hk.

Table of Contents

		Page no.
1	Crystallographic data, X-ray structures and selected bond lengths and angles	S2
2	NMR spectra	S9
3	Mass spectra	S30
4	Peroxide test	S31
5	IR spectrum	S31

1. Crystallographic data and crystal structures

Table S1. Crystallographic Data and Refinement Details for Complexes **2, 4, and 6-8**

Compound	**2**	**6**	**7·H$_2$O**	**8**	**4**
Empirical formula	$C_{38}H_{76}CeCo_2O_{22}P_6$	$C_{42}H_{77}CeCo_2O_{20}P_6$	$C_{34}H_{76}CeCo_2N_2O_{25}P_6S_2$	$C_{38}H_{72}CeCo_2F_6N_2O_{20}P_6$	$C_{48}H_{78}CeCo_2N_2O_{26}P_6$
Formula weight	1328.78	1345.83	1420.88	1434.77	1542.92
Temperature/K	173.01(10)	173.00(10)	173.00(10)	170(4)	173.00(10)
Crystal system	monoclinic	monoclinic	monoclinic	monoclinic	orthorhombic
Space group	$P2_1/n$	$P2_1/c$	$P2_1/n$	I2/a	$P2_12_12_1$
a/Å	12.2041(3)	12.1287(2)	18.0772(3)	24.0140(5)	13.07759(14)
b/Å	23.4560(4)	18.1071(2)	17.4608(3)	23.4029(4)	20.0828(2)
c/Å	19.6859(5)	26.3465(3)	18.4648(3)	23.2666(5)	24.7647(3)
α/°	90	90	90	90	90
β/°	90.458(2)	91.8510(10)	97.6430(10)	114.845(3)	90
γ/°	90	90	90	90	90
Volume/Å^3	5635.1(2)	5783.08(13)	5776.49(17)	11865.6(5)	6504.09(12)
Z	4	4	4	8	4
ρ_{calc}g/cm^3	1.566	1.546	1.634	1.606	1.576
μ/mm^{-1}	12.914	12.567	13.349	12.450	11.340
F(000)	2728.0	2764.0	2912.0	5840.0	3160.0
Crystal size/mm^3	0.2 × 0.15 × 0.1	0.16 × 0.15 × 0.05	0.08 × 0.08 × 0.08	0.13 × 0.13 × 0.12	0.13 × 0.09 × 0.07
Radiation	CuKα (λ = 1.54184)	CuKα (λ = 1.54184)	CuKα (λ = 1.54184)	CuKα (λ = 1.54184)	CuKα (λ = 1.54184)
2Θ range /°	5.862 to 154.406	5.924 to 148.488	6.428 to 153.778	5.542 to 148.524	5.666 to 154.514
Index ranges	-15 ≤ h ≤ 15, -25 ≤ k ≤ 29, -15 ≤ l ≤ 24	-15 ≤ h ≤ 14, -21 ≤ k ≤ 22, -32 ≤ l ≤ 22	-22 ≤ h ≤ 22, -20 ≤ k ≤ 21, -21 ≤ l ≤ 23	-28 ≤ h ≤ 29, -29 ≤ k ≤ 18, -29 ≤ l ≤ 28	-12 ≤ h ≤ 16, -25 ≤ k ≤ 20, -28 ≤ l ≤ 30
Reflections collected	36311	34612	37845	34852	42892
Independent reflections	11693 [R_{int} = 0.0573, R_{sigma} = 0.0558]	11530 [R_{int} = 0.0357, R_{sigma} = 0.0361]	12025 [R_{int} = 0.0517, R_{sigma} = 0.0550]	11858 [R_{int} = 0.0500, R_{sigma} = 0.0533]	13498 [R_{int} = 0.0461, R_{sigma} = 0.0480]
Data/restraints/parameters	11693/92/718	11530/776/904	12025/40/691	11858/313/734	13498/195/901
Goodness-of-fit on F^2	1.012	1.018	1.041	1.036	1.020
Final R indexes [I>=2σ (I)]	R_1 = 0.0521, wR_2 = 0.1308	R_1 = 0.0473, wR_2 = 0.1264	R_1 = 0.0520, wR_2 = 0.1427	R_1 = 0.0554, wR_2 = 0.1415	R_1 = 0.0342, wR_2 = 0.0704
Final R indexes [all data]	R_1 = 0.0682, wR_2 = 0.1410	R_1 = 0.0595, wR_2 = 0.1361	R_1 = 0.0672, wR_2 = 0.1545	R_1 = 0.0783, wR_2 = 0.1598	R_1 = 0.0421, wR_2 = 0.0737
Largest diff. peak/hole/e Å^{-3}	0.80/-0.73	1.37/-0.70	1.96/-1.21	1.39/-1.11	0.39/-0.79

Disorder in crystal structures

In **2**, the Ce1 atom was split into two positions with occupancy of 0.89 and 0.11. The O10, O17-O18 and P4-P6 atoms of one L_{OEt} group were each split two positions with occupancy of 0.89 and 0.11. The C21-C22 atoms of the ethoxy group were split into two positions with occupancy of 0.72 and 0.28. In **6**, the O6, O2-O3, O11-O16, C13-C22 and C31-42 atoms of the ethoxy groups are 50:50 disordered, respectively. In **7**, the O4 and C17-C18 atoms of the ethoxy group were split into two positions with occupancy of 0.75 and 0.25. The unit cell of **8** contains two independent molecules. In one of these molecules, the C1A-C5A atoms of the Cp ring are disordered, and each atom was refined with two positions with occupancy of 0.50 and 0.50. In **4**, the N2 and O32-O33 atoms of the 2-nitrobenzoate group are 50:50 disordered, respectively. The C36 atom of the ethoxy group was refined with two positions with occupancy of 0.75 and 0.25. The C13-C14, C17-C18, C21-C22 and C31-C32 atoms of the ethoxy group are 50:50 disordered.

References for X-ray crystallography

1. Dolomanov, O.V., Bourhis, L.J., Gildea, R.J, Howard, J.A.K. & Puschmann, H. (2009), *J. Appl. Cryst*. **42**, 339-341.
2. Sheldrick, G.M. (2015). *Acta Cryst. A71*, 3-8.
3. Sheldrick, G.M. (2015). *Acta Cryst. C71*, 3-8.

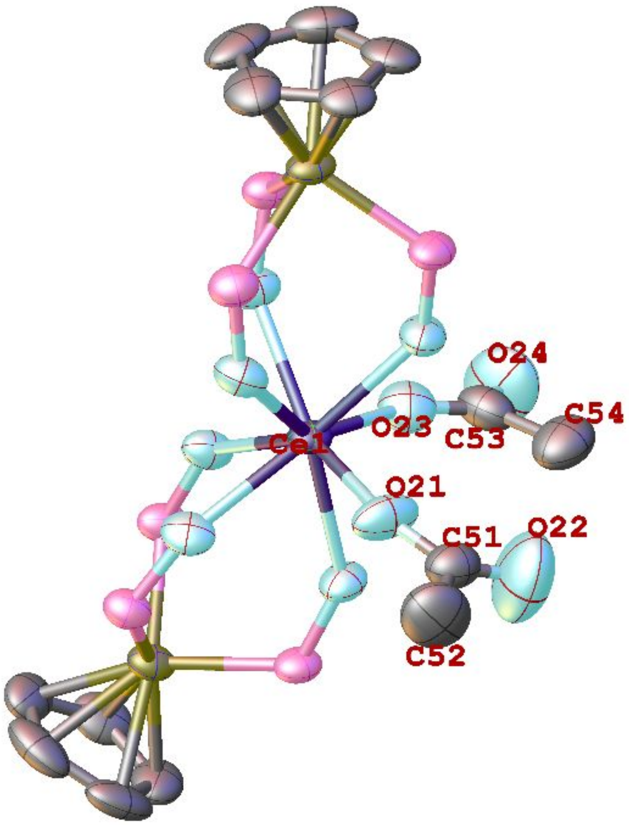

Figure S1. Molecular structure of [Ce(L_{OEt})$_2$(CH_3CO_2)$_2$] (**2**). Hydrogen atoms and ethoxy groups of L_{OEt}^- ligands are omitted for clarity. The ellipsoids are drawn at 40% probability level.

Table S2. Selected bond lengths (Å) and angles (°) for **2**

Ce1-O23	2.226(5)	Ce1-O21	2.260(5)
O23-C53	1.265(8)	O21-C51	1.268(8)
Ce1-O23-C53	152.0(5)	Ce1-O21-C51	151.1(5)
Ce1-O(L_{OEt})	2.346(4)-2.367(4)	O21-Ce1-O23	114.2(6)

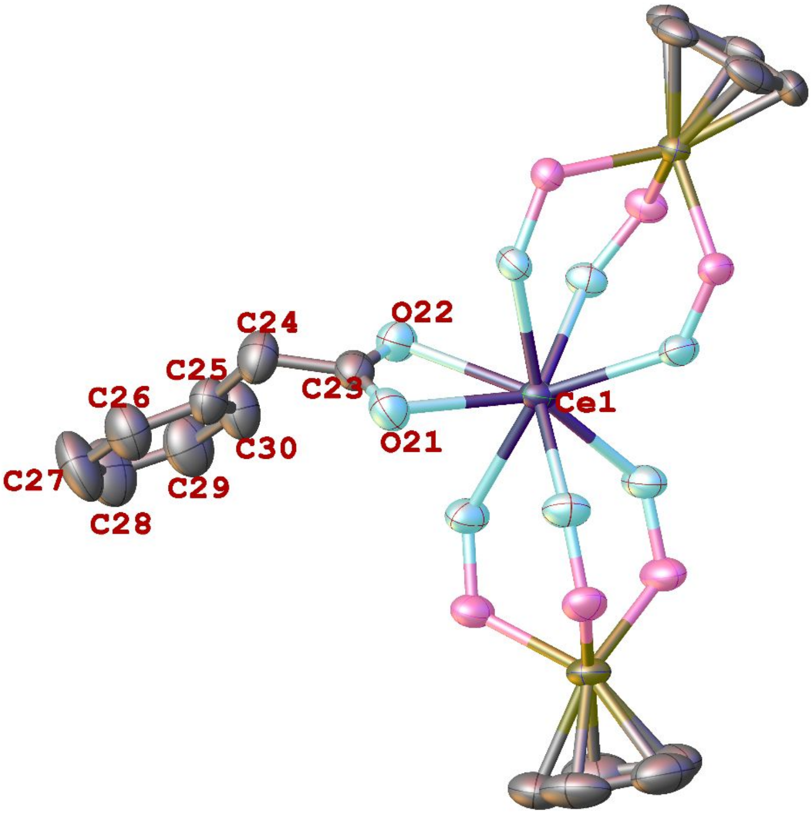

Figure S2. Molecular structure of [$Ce^{III}(L_{OEt})_2(PhCH_2CO_2)$] (**6**). Hydrogen atoms and ethoxy groups of L_{OEt}^- ligands are omitted for clarity. The ellipsoids are drawn at 40% probability level.

Table S3. Selected bond lengths (Å) and angles (°) for **6**

Ce1-O21	2.572(4)	Ce1-O22	2.531(4)
O21-C23	1.273(7)	O22-C23	1.262(7)
C23-O21-Ce1	92.0(3)	C23-O22-Ce1	94.2(3)
Ce1-O(L_{OEt})	2.409(3)- 2.511(3)	O22-Ce1-O21	51.55(12)

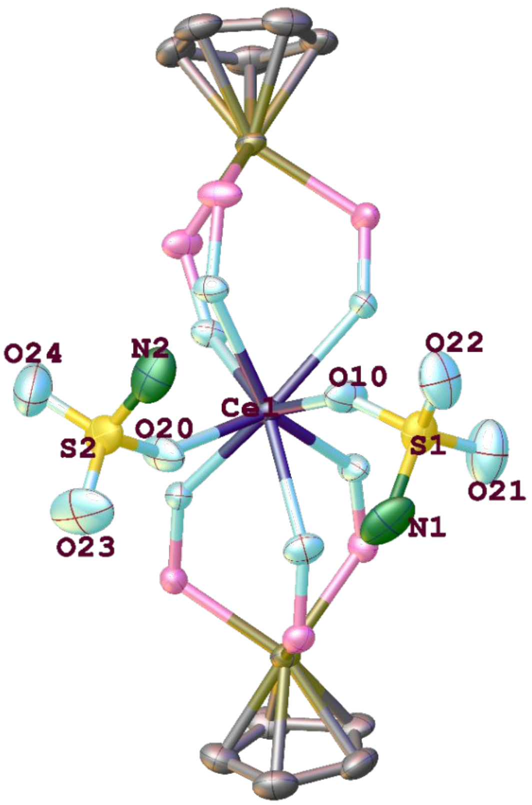

Figure S3. Molecular structure of $[Ce(L_{OEt})_2(SO_3NH_2)_2]$ (**7**). Hydrogen atoms, co-crystallized H-bonded H_2O molecule and ethoxy groups of L_{OEt}^- ligands are omitted for clarity. The ellipsoids are drawn at 40% probability level.

Table S4. Selected bond lengths (Å) and angles (°) for **7**

Ce1-O10	2.349(4)	Ce1-O20	2.312(4)
O10-S1	1.482(4)	O20-S2	1.474(4)
S1-O10-Ce1	141.0(3)	S2-O20-Ce1	150.6(3)
Ce1-O(L_{OEt})	2.273(3)- 2.384(3)	O20-Ce1-O10	82.93(18)
S1-O21	1.430(5)	S2-O23	1.432(6)
S1-O22	1.426(6)	S2-O24	1.419(6)
S1-N1	1.629(7)	S2-N2	1.618(7)

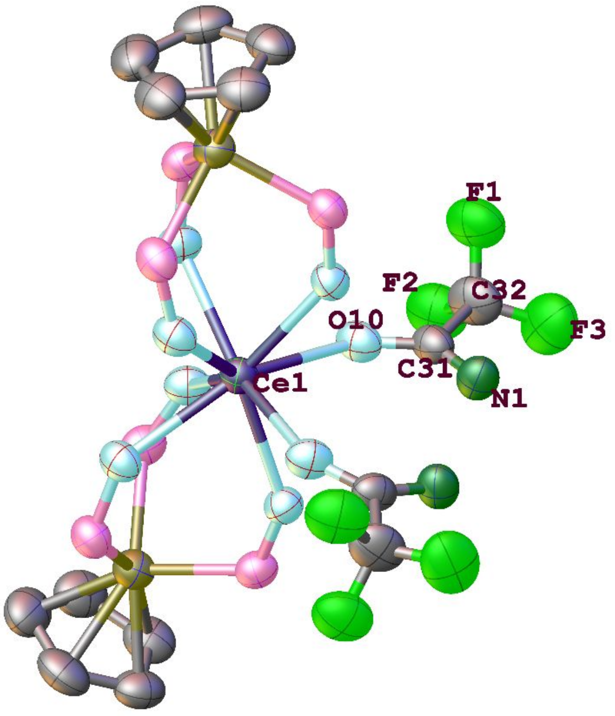

Figure S4. Molecular structure of one of the two asymmetric units of $[Ce(L_{OEt})_2(CF_3CONH)_2]$ (**8**). Hydrogen atoms and ethoxy groups of L_{OEt}^- ligands are omitted for clarity. The ellipsoids are drawn at 40% probability level.

Table S5. Selected bond lengths (Å) and angles (°) for **8**

Ce1-O10	2.582(4)	Ce1-O10-C31	138.9(4)
O10-C31	1.238(7)	O10-C31-N1	129.3(6)
C31-N1	1.251(8)	O10-Ce1-O10’	100.6(2)
Ce1-O(L_{OEt})	2.446(4)- 2.453(4)		

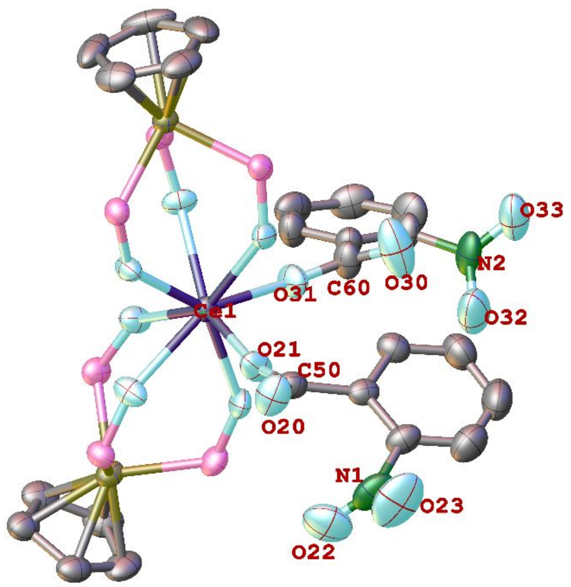

Figure S5. Molecular structure of [Ce(L_{OEt})$_2$(2-$NO_2C_6H_4CO_2$)$_2$] (**4**). Hydrogen atoms and ethoxy groups of L_{OEt}^- ligands are omitted for clarity. The ellipsoids are drawn at 40% probability level.

Table S6. Selected bond lengths (Å) and angles (°) for **4**

Ce1-O21	2.235(4)	Ce1-O31	2.255(4)
O21-C50	1.276(7)	O31-C60	1.273(8)
Ce1-O21-C50	164.9(5)	Ce1-O31-C60	165.3(5)
Ce1-O(L_{OEt})	2.345(4)-2.372(4)	O21-Ce1-O31	99.73(17)

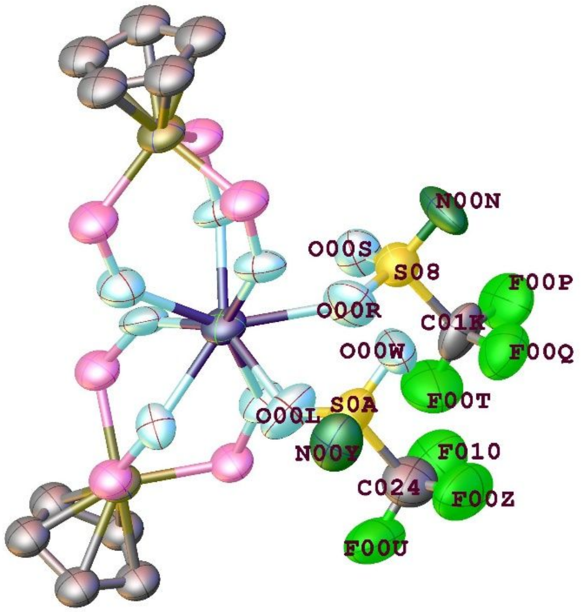

Figure S6. Preliminary X-ray structure of $[Ce(L_{OEt})_2(CF_3SO_2NH)_2]$ (**9**). Hydrogen atoms and ethoxy groups of L_{OEt}^- ligands are omitted for clarity. The ellipsoids are drawn at 40% probability level.

2. NMR spectra

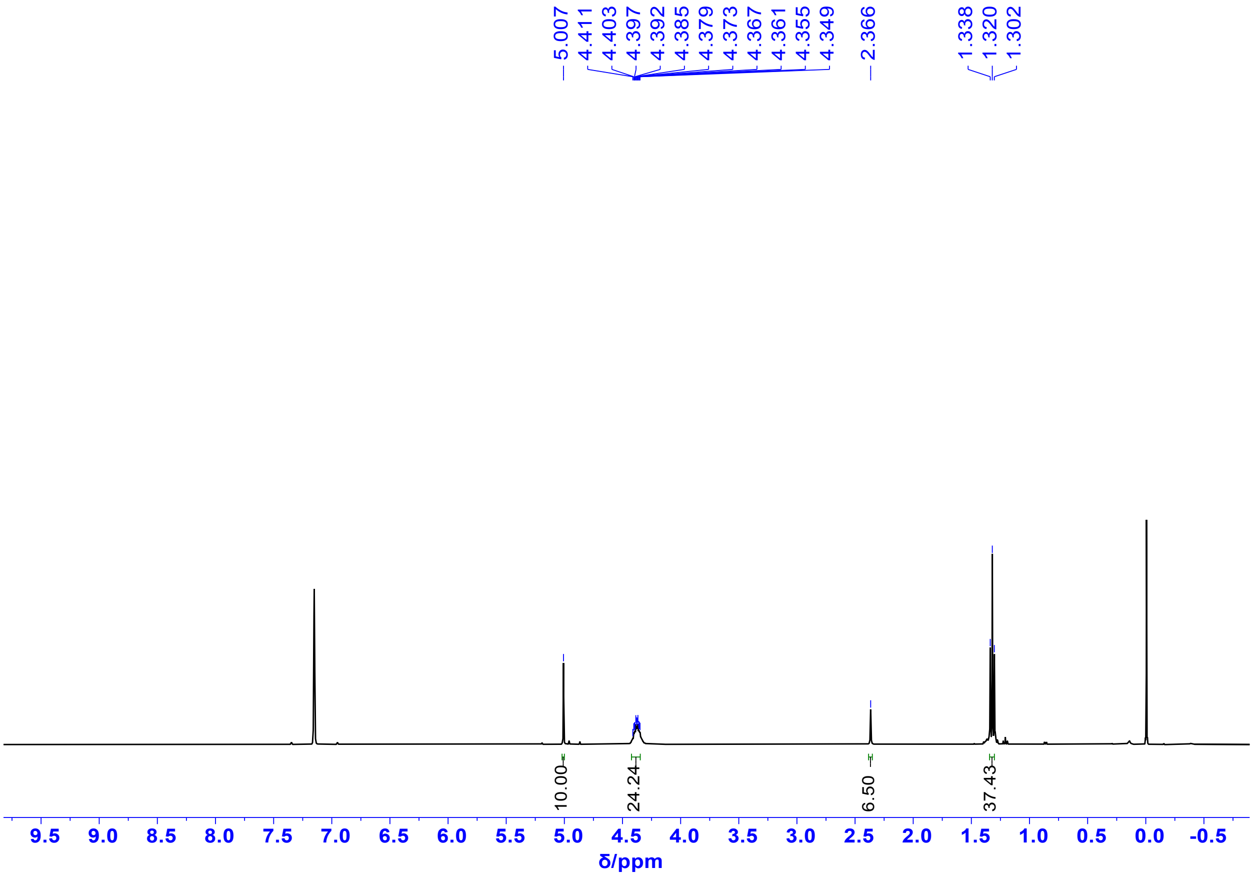

Figure S7a. ^{1}H NMR spectrum (400 MHz, C_6D_6, 25 ^oC) of $[Ce(L_{OEt})_2(CH_3CO_2)_2]$ (**2**)

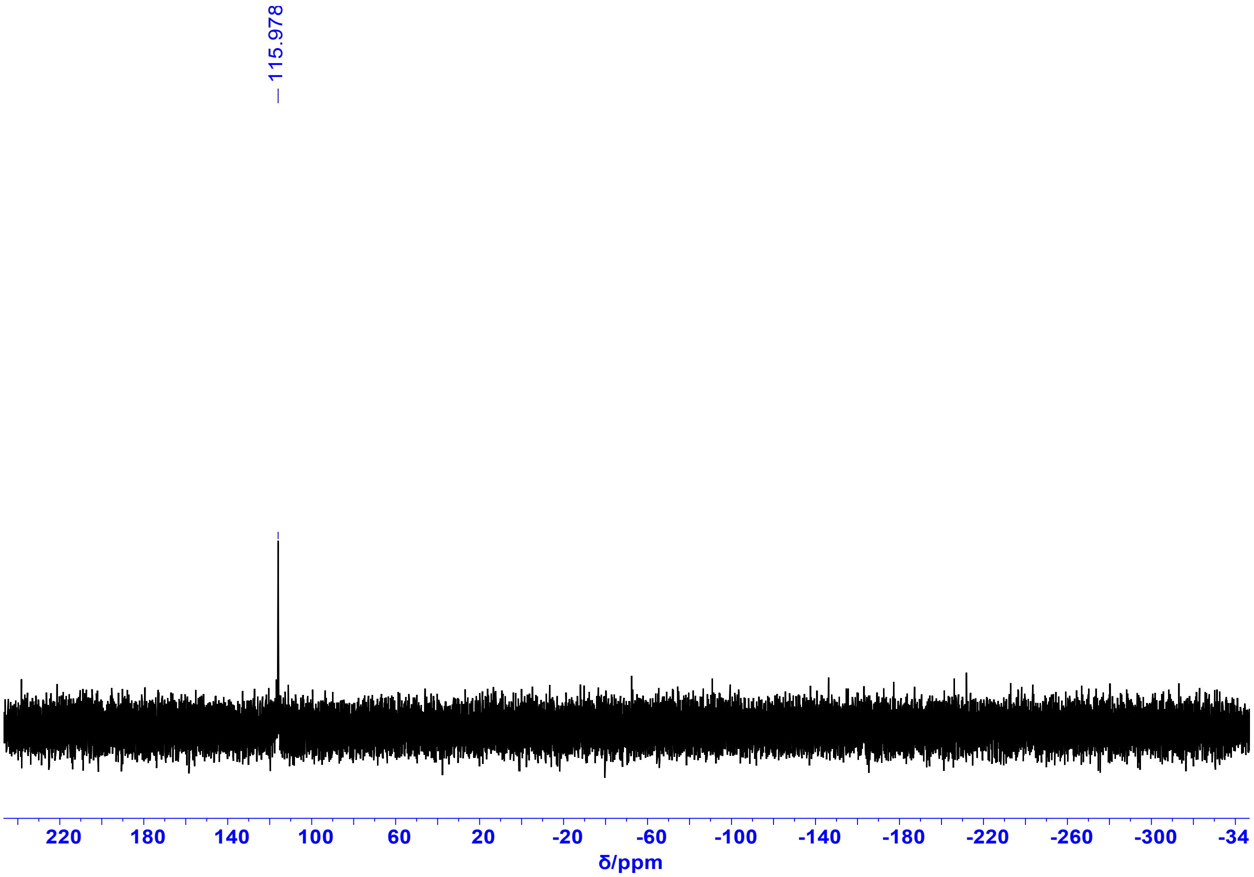

Figure S7b. $^{31}P\{^{1}H\}$ NMR spectrum (162 MHz, C_6D_6, 25 ^{o}C) of $[Ce(L_{OEt})_2(CH_3CO_2)_2]$ (**2**)

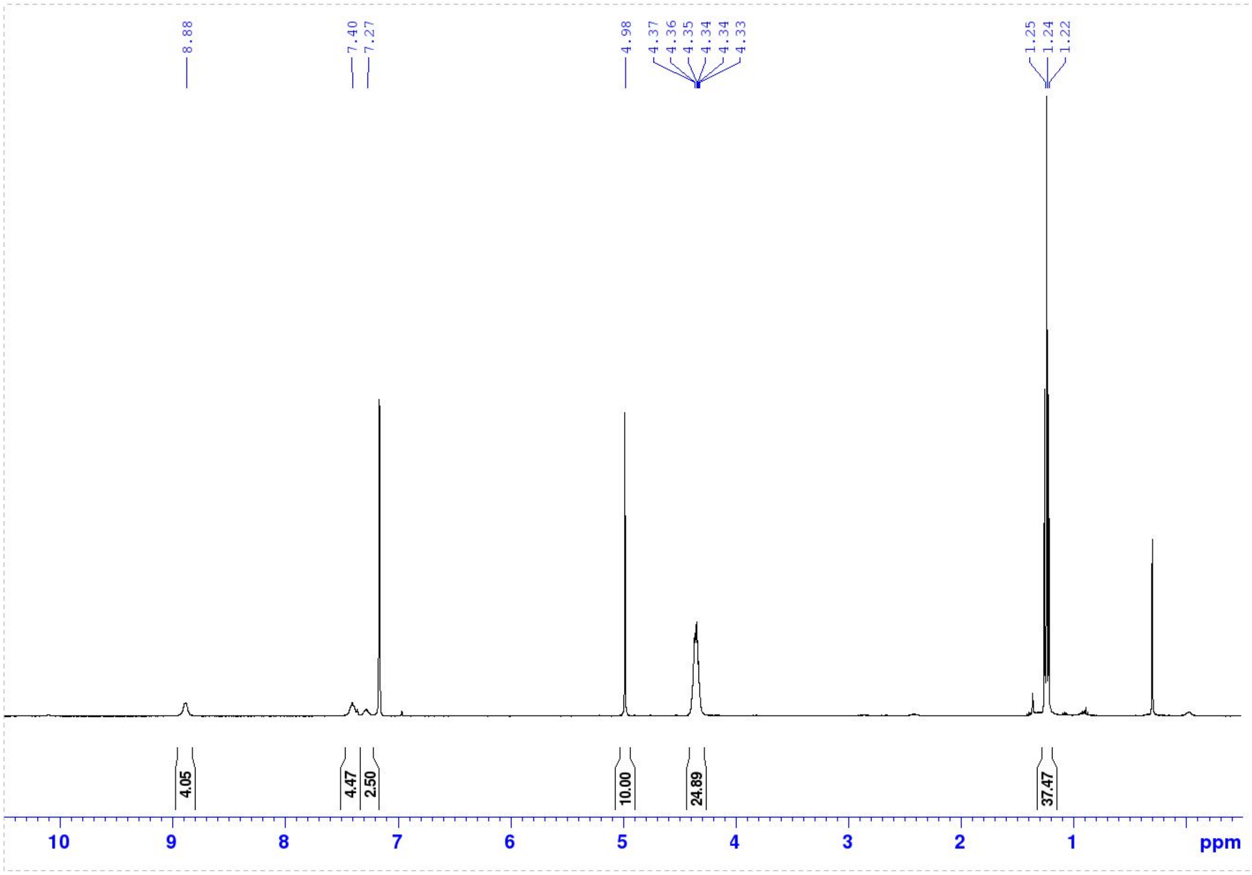

Figure S8a. ^{1}H NMR spectrum (400 MHz, C_6D_6, 25 ^oC) of $[(L_{OEt})_2Ce(PhCO_2)_2]$ **(3)**

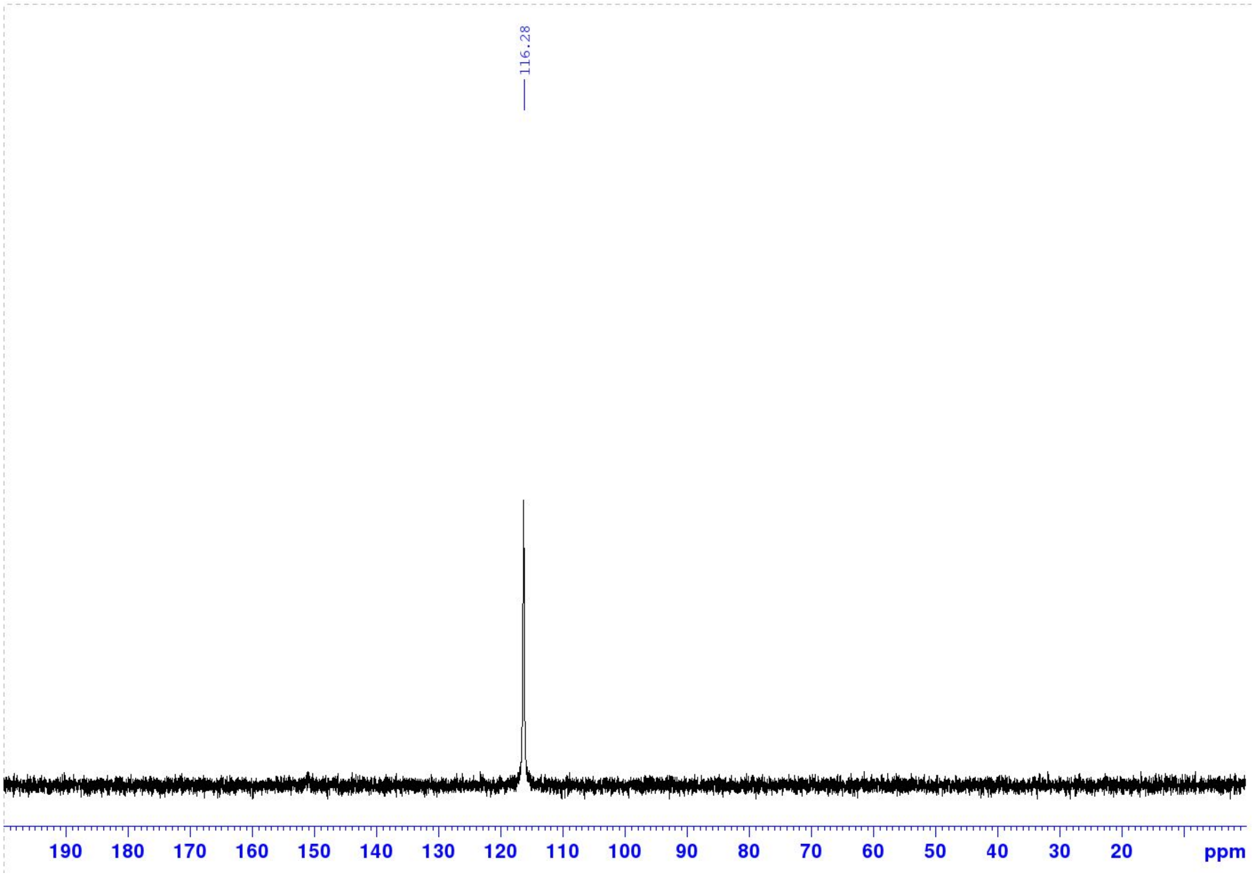

Figure S8b. $^{31}P\{^{1}H\}$ NMR spectrum (162 MHz, C_6D_6, 25 ^{o}C) of $[(L_{OEt})_2Ce(PhCO_2)_2]$ **(3)**

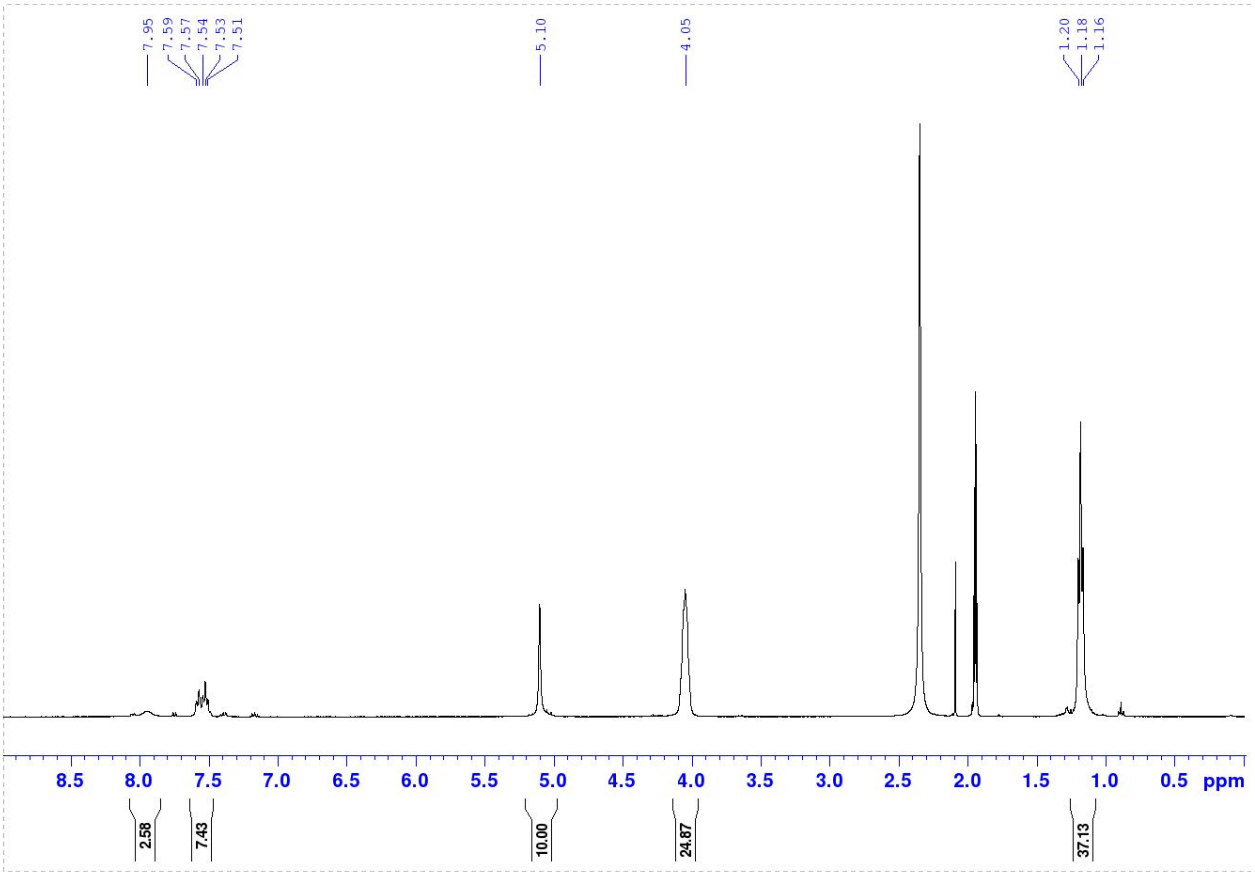

Figure S9a. ^1H NMR spectrum (400 MHz, CD_3CN, 25 ^oC) of $[Ce(L_{OEt})_2(2\text{-}NO_2C_6H_4CO_2)_2]$ **(4)**

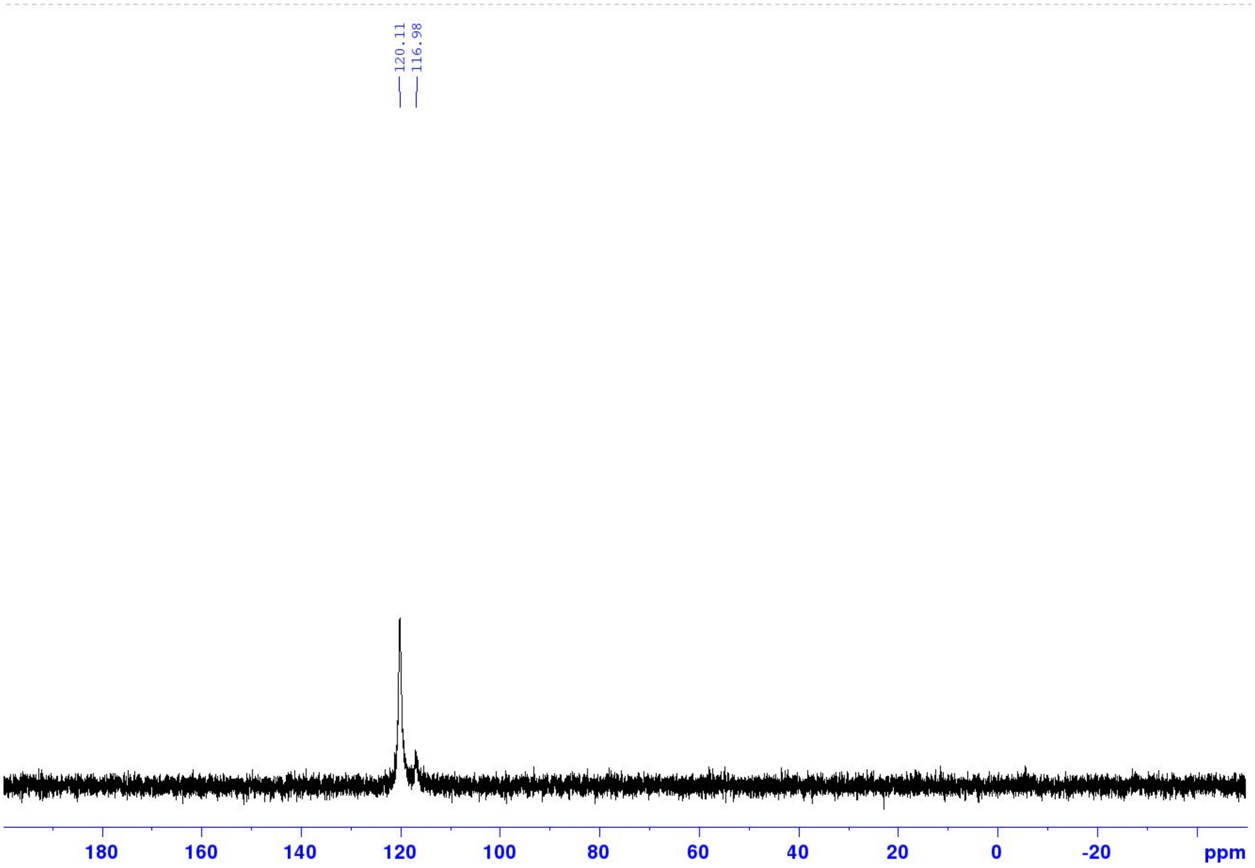

Figure S9b. $^{31}P\{^{1}H\}$ NMR spectrum (162 MHz, CD_3CN, 25 °C) of $[Ce(L_{OEt})_2(2\text{-}NO_2C_6H_4CO_2)_2]$ **(4)**

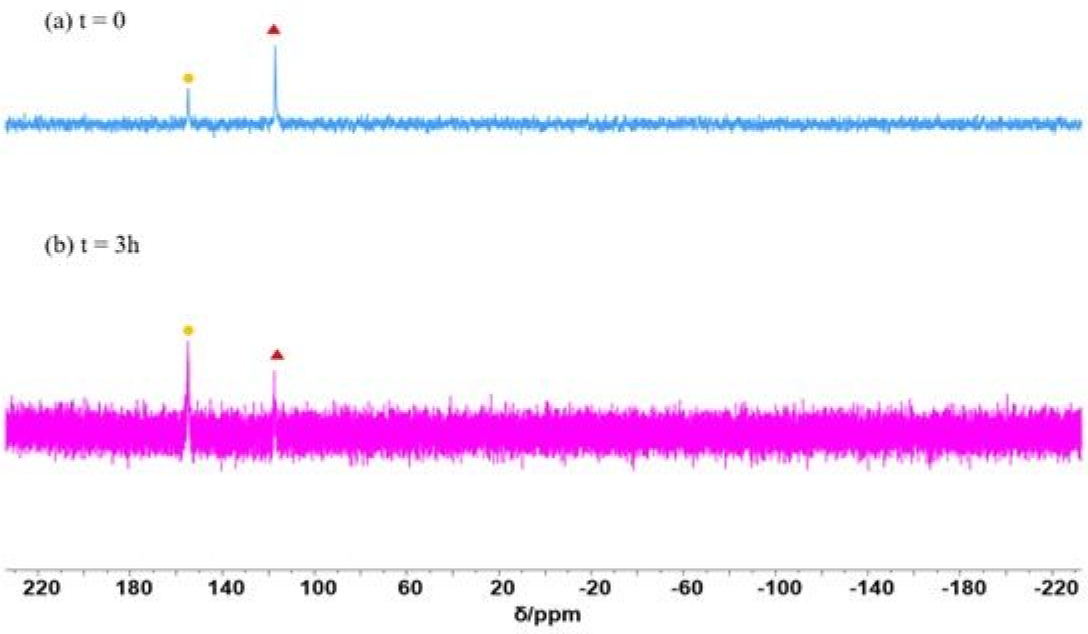

Figure S10. $^{31}P\{^{1}H\}$ NMR spectra (162 MHz, C_6D_6, 25 °C) of a mixture of $[Ce^{IV}(L_{OEt})_2(O_2CCH_2Ph)_2]$ (**5**) and $[Ce^{III}(L_{OEt})_2(O_2CCH_2Ph)]$ (**6**) in benzene under N_2 in the dark at t = 0 (Ce(IV): Ce(III) ~ 3:1) (a) and 3 h (Ce(IV): Ce(III) ~ 1:2) (b). ● = Ce(III), ▲= Ce(IV).

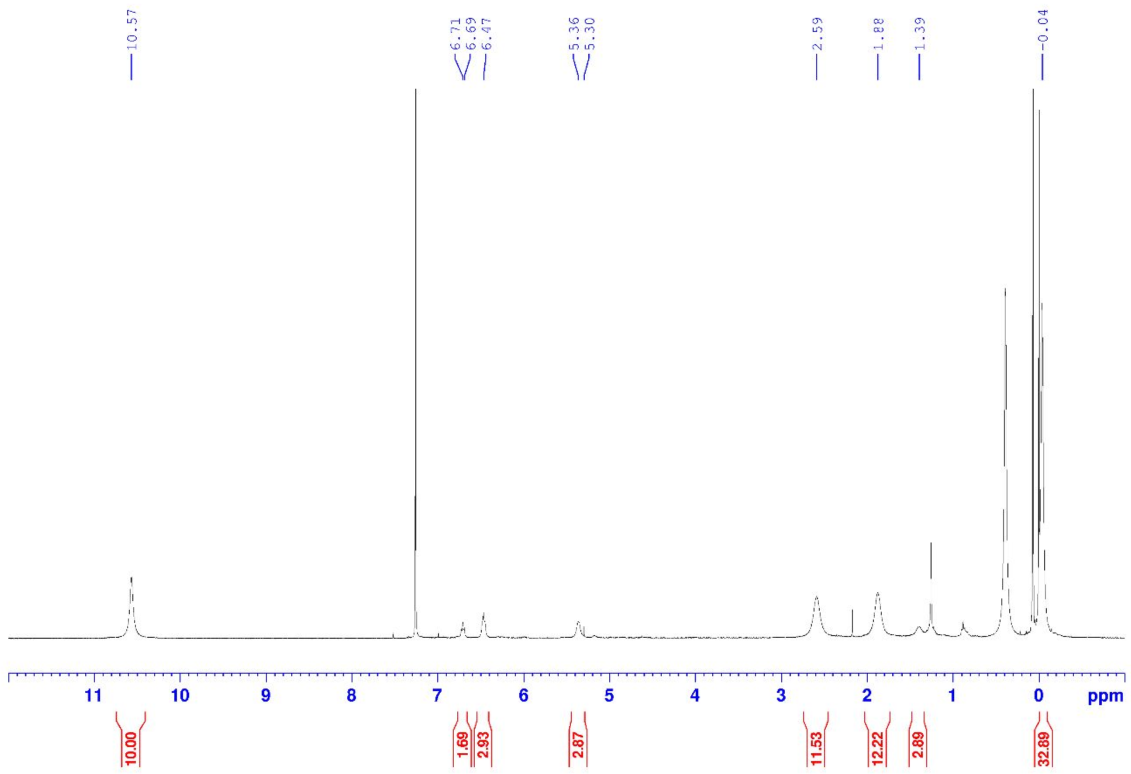

Figure S11a. ^{1}H NMR spectrum (400 MHz, $CDCl_3$, 25 ^{o}C) of $[Ce^{III}(L_{OEt})_2(O_2CCH_2Ph)]$ **(6)**

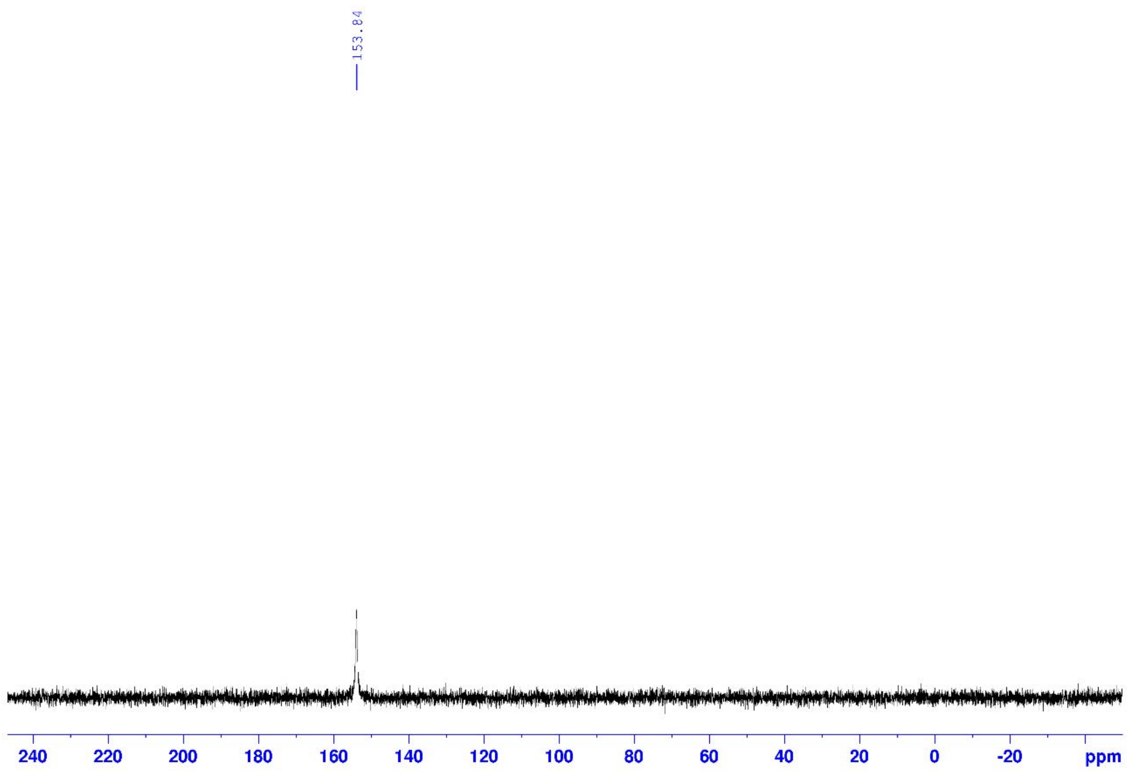

Figure S11b. $^{31}P\{^{1}H\}$ NMR spectrum (162 MHz, $CDCl_3$, 25 °C) of $[Ce^{III}(L_{OEt})_2(O_2CCH_2Ph)]$ **(6)**

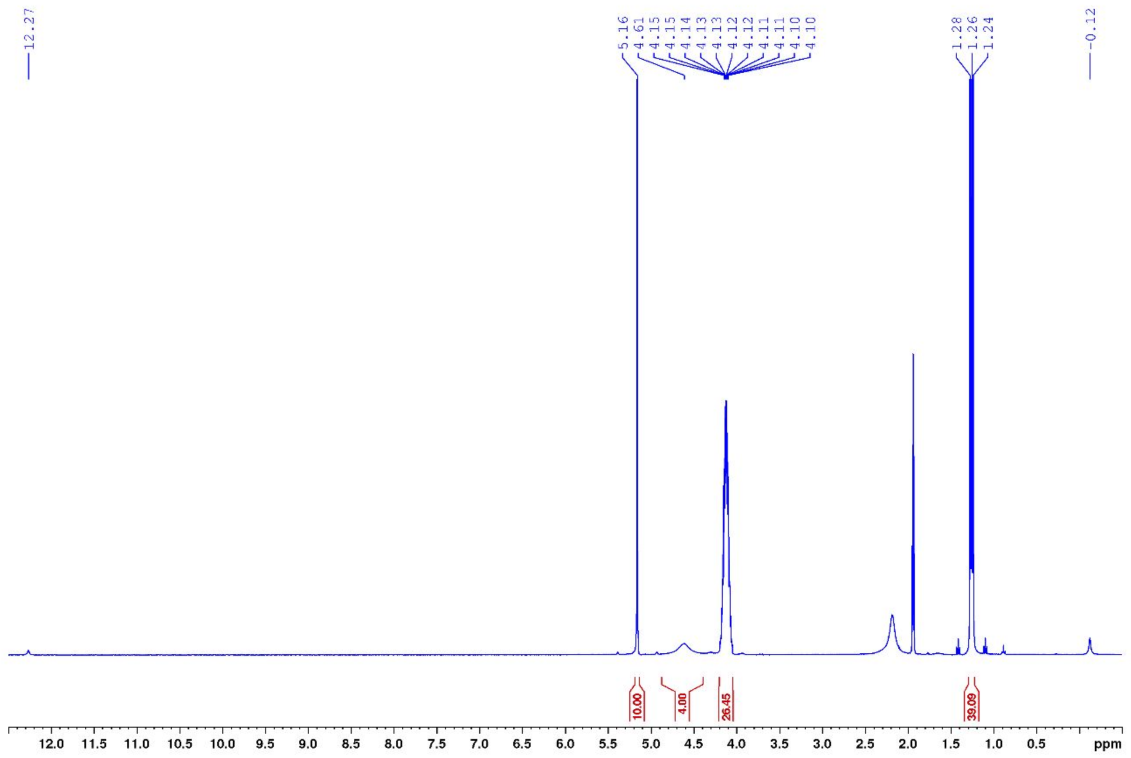

Figure S12a. ^{1}H NMR spectrum (400 MHz, CD_3CN, 25°C) of $[Ce(L_{OEt})_2(SO_3NH_2)_2]$ (**7**)

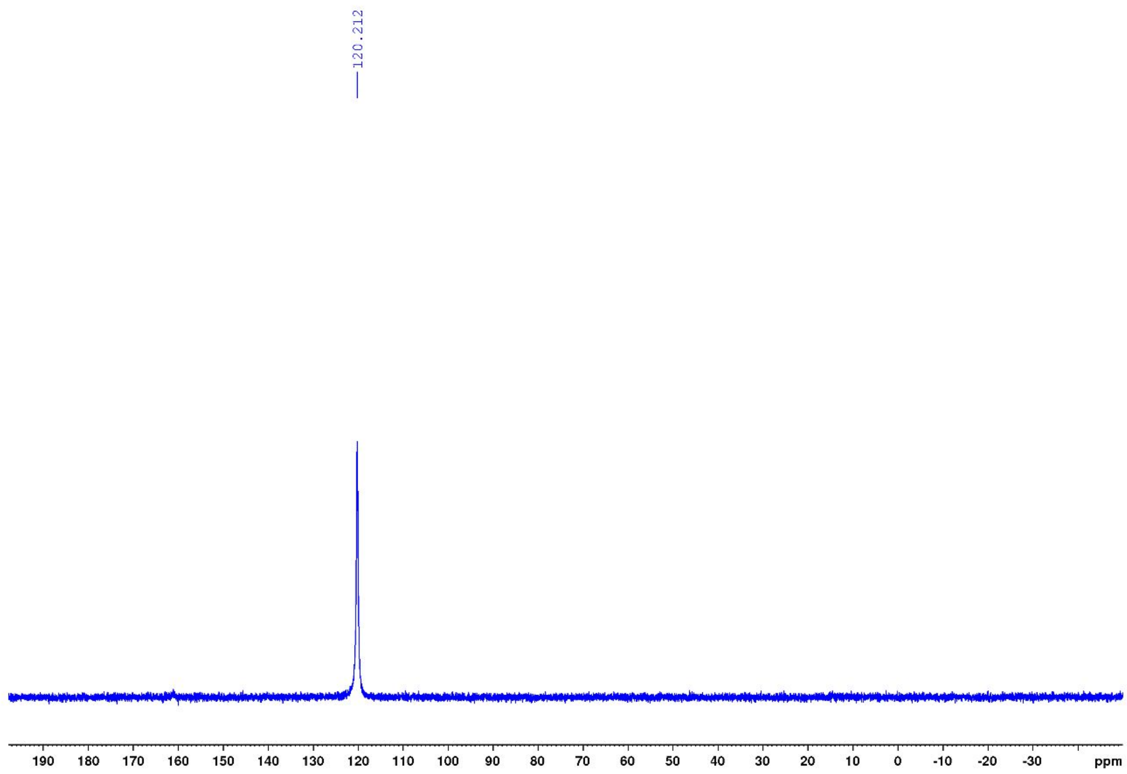

Figure S12b. $^{31}P\{^{1}H\}$ NMR (162 MHz, CD_3CN, 25°C) of $[Ce(L_{OEt})_2(SO_3NH_2)_2]$ (**7**)

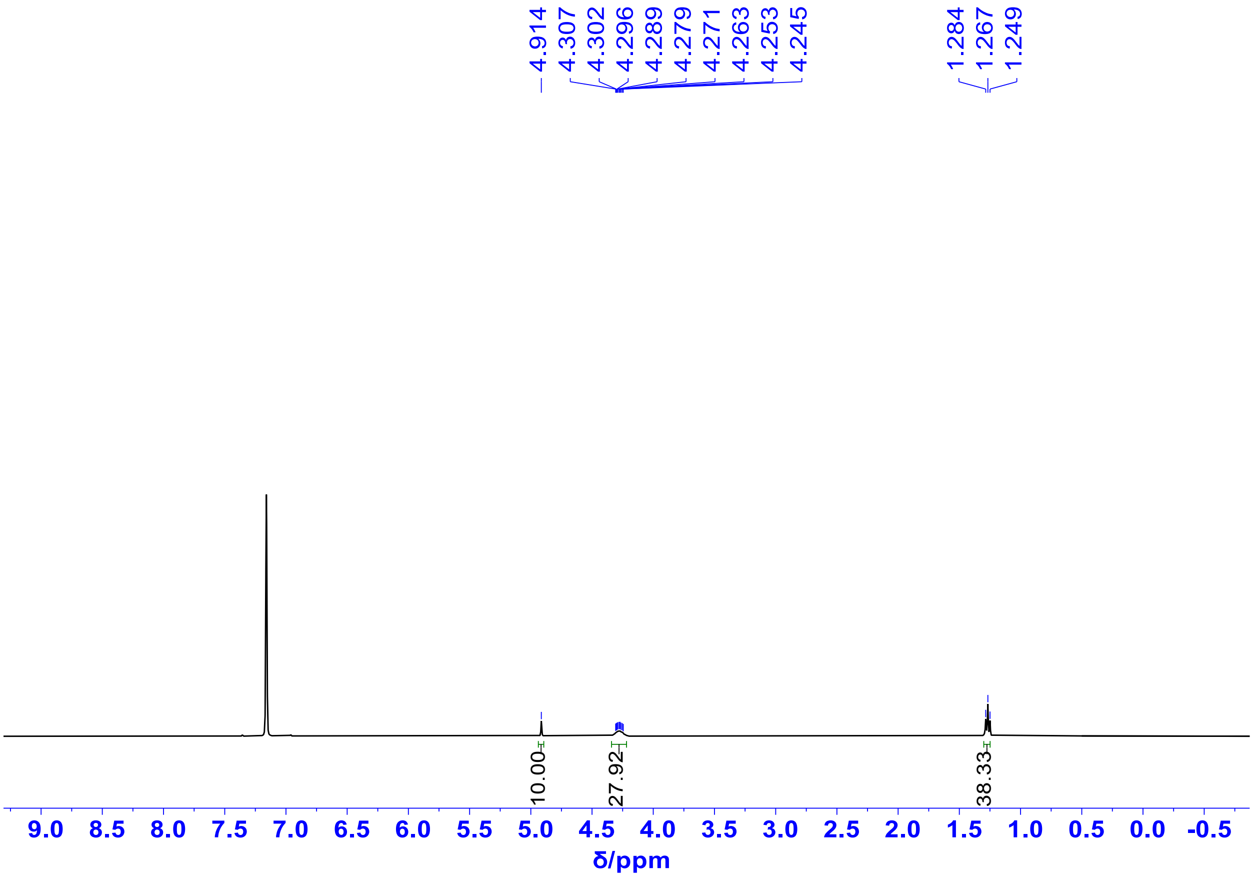

Figure S13a. ^{1}H NMR spectrum (400 MHz, C_6D_6, 25 °C) of $[Ce(L_{OEt})_2(CF_3CONH)_2]$ **(8)**

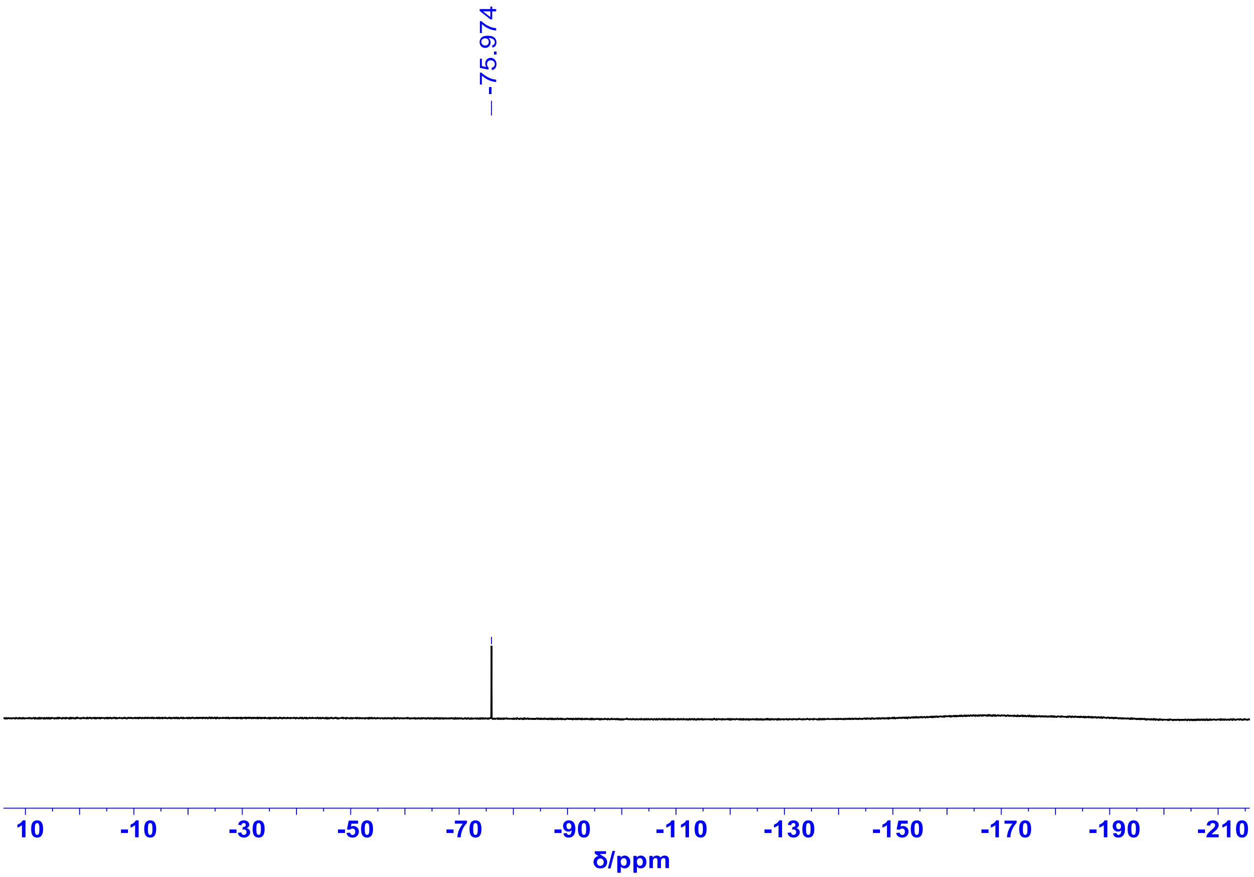

Figure S13b. $^{19}F\{^{1}H\}$ NMR spectrum (376 MHz, C_6D_6, 25 °C) of $[Ce(L_{OEt})_2(CF_3CONH)_2]$ **(8)**

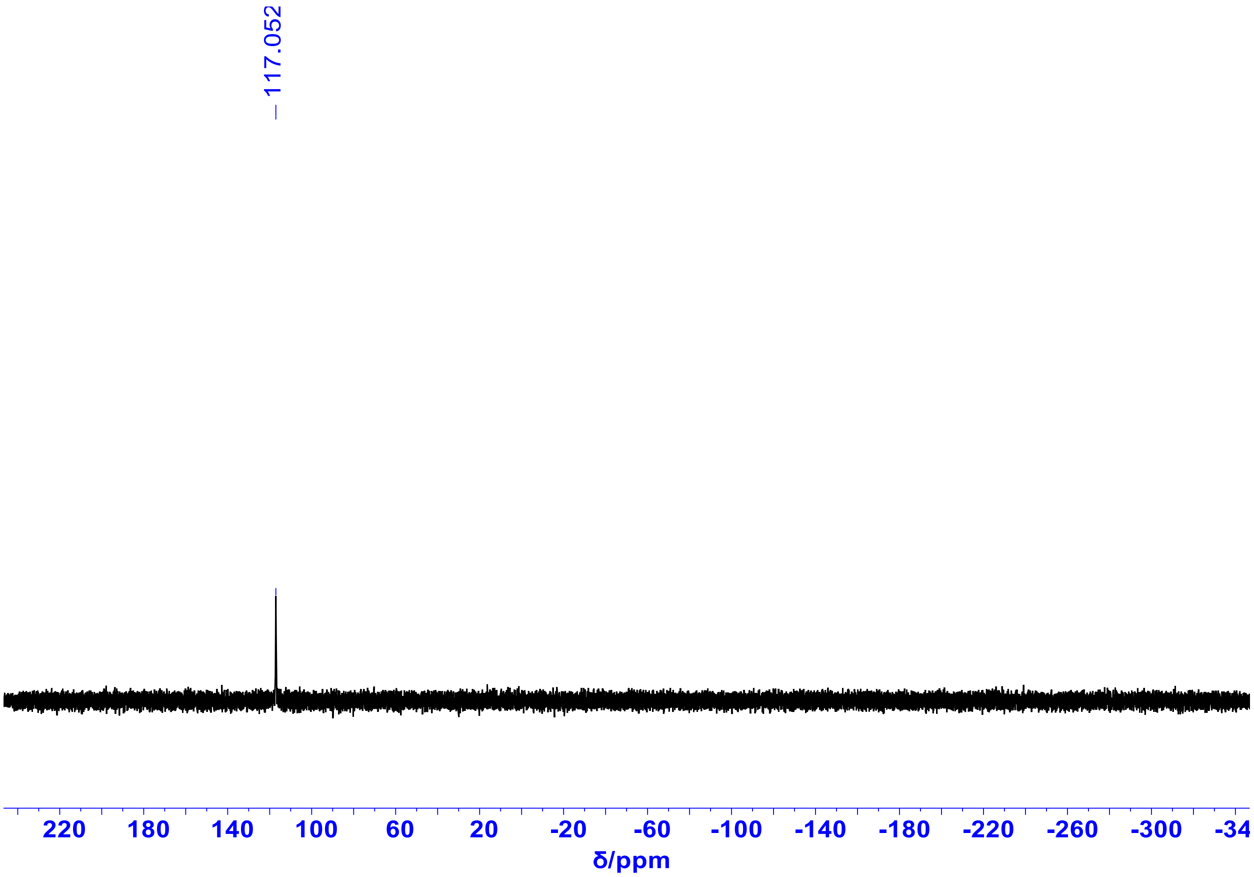

Figure S13c. $^{31}P\{^{1}H\}$ NMR spectrum (162 MHz, C_6D_6, 25 ^{o}C) of $[Ce(L_{OEt})_2(CF_3CONH)_2]$ **(8)**

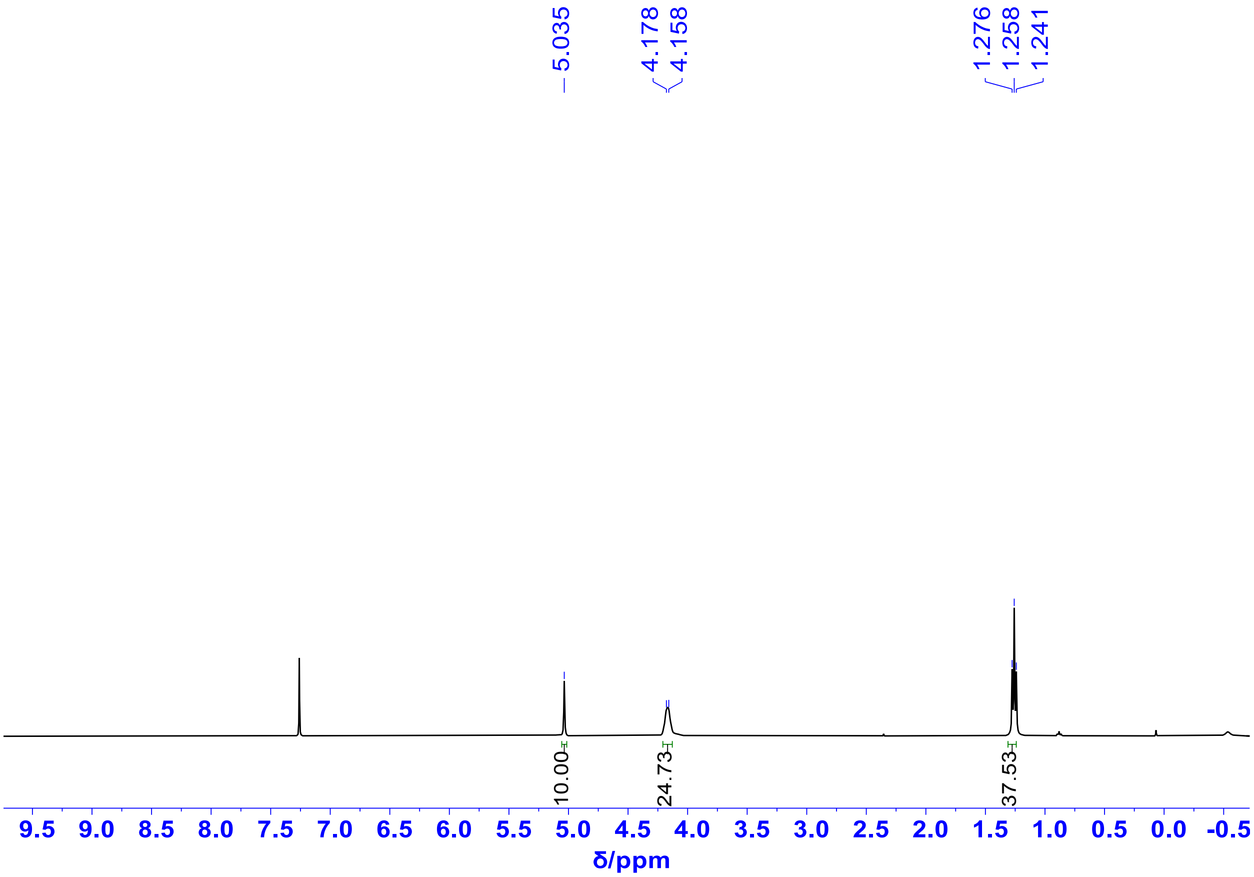

Figure S14a. ^{1}H NMR spectrum (400 MHz, $CDCl_3$, 25 ^{o}C) of $[Ce(L_{OEt})_2(CF_3SO_2NH)_2]$ **(9)**

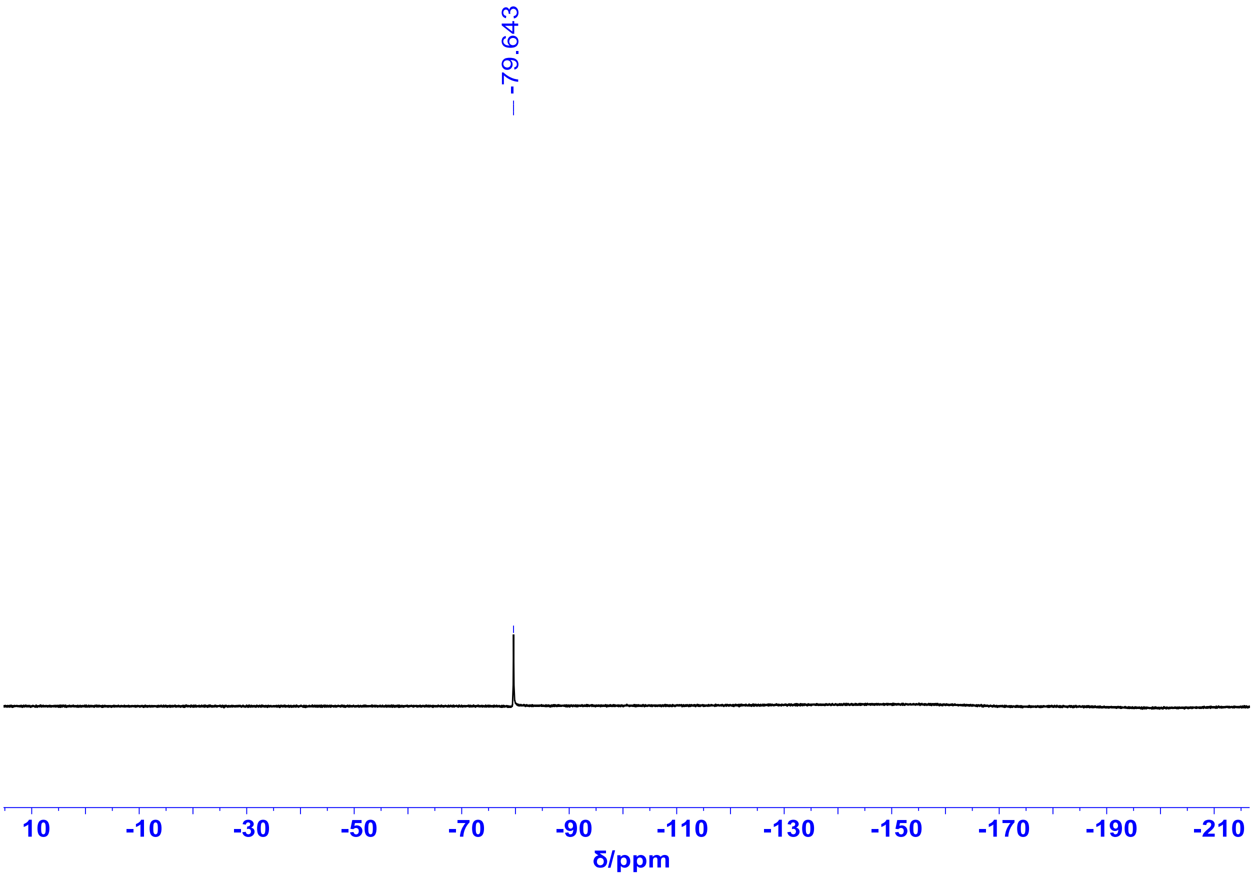

Figure S14b. $^{19}F\{^{1}H\}$ NMR spectrum (376 MHz, $CDCl_3$, 25 °C) of $[Ce(L_{OEt})_2(CF_3SO_2NH)_2]$ **(9)**

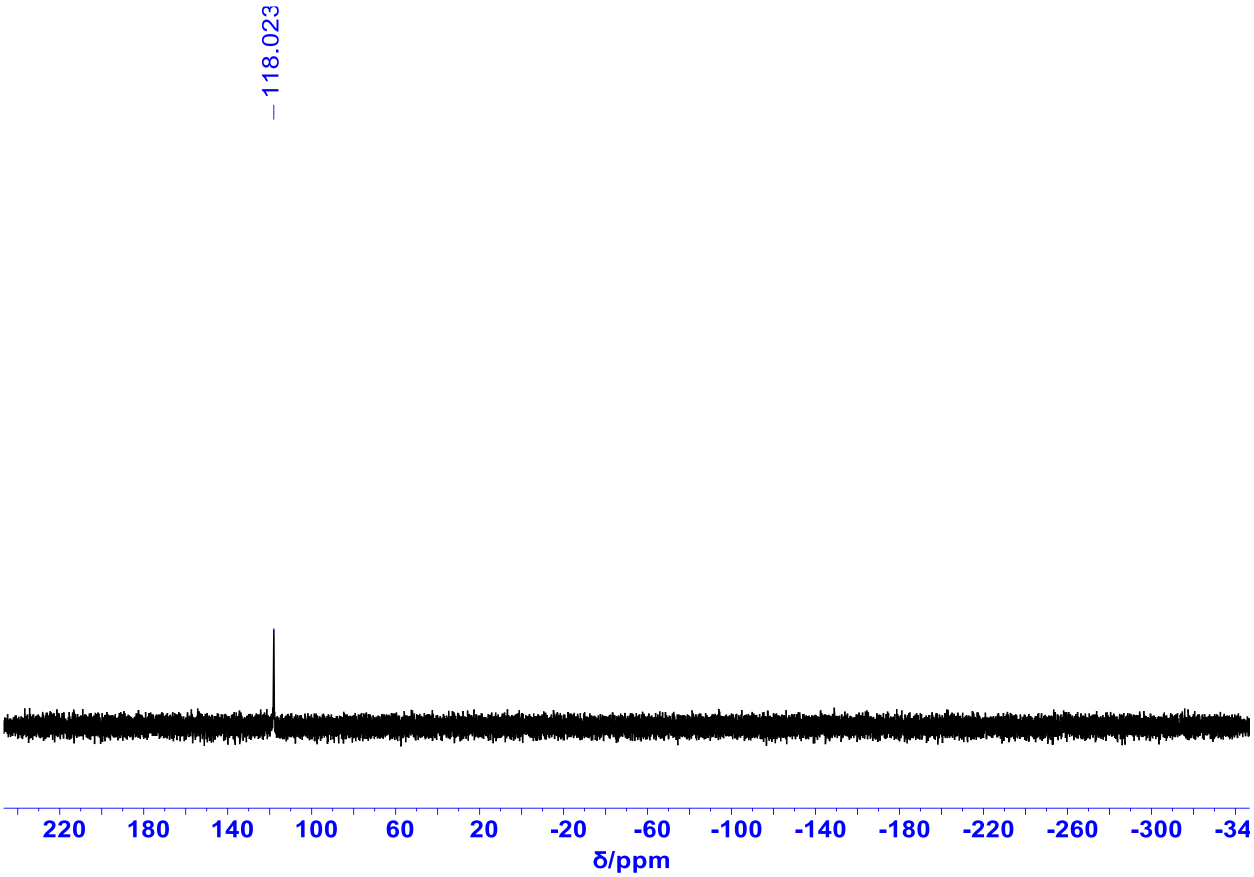

Figure S14c. $^{31}P\{^{1}H\}$ NMR spectrum (162 MHz, $CDCl_3$, 25 °C) of $[Ce(L_{OEt})_2(CF_3SO_2NH)_2]$ **(9)**

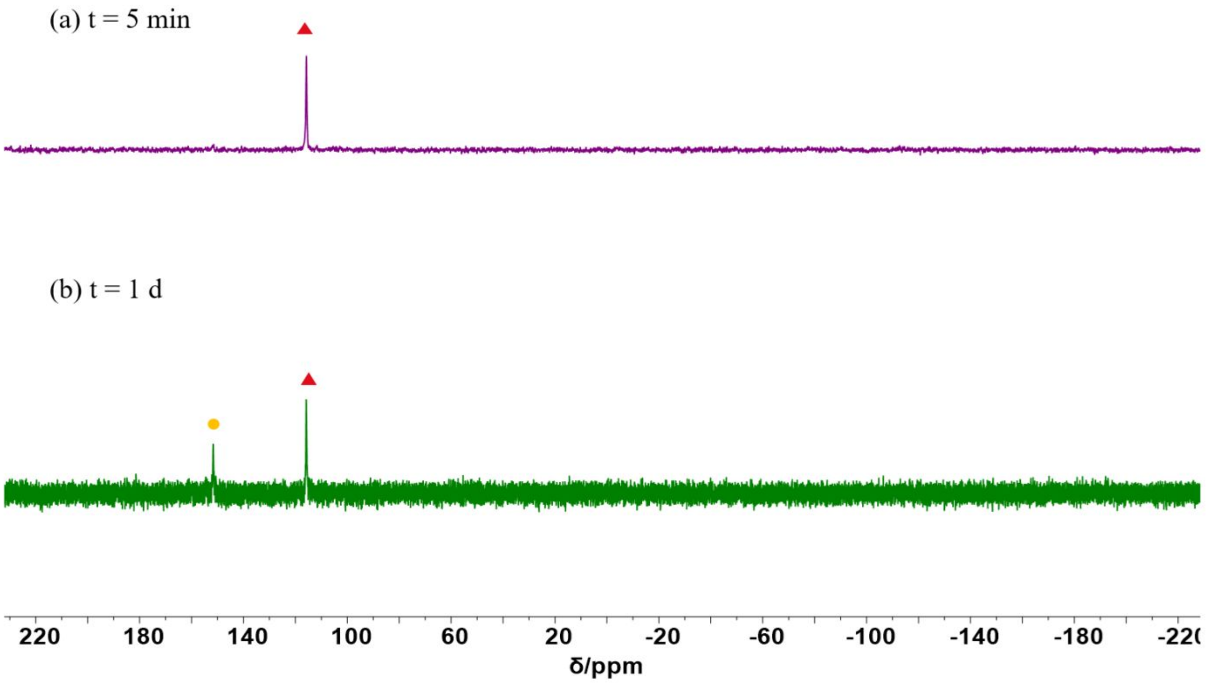

Figure S15. $^{31}P\{^{1}H\}$ NMR spectra (162 MHz , 25 ^{o}C) of $[(L_{OEt})_2Ce(CO_3)]$ in distilled THF under N_2 at t = 5 min (a) and t = 1 day (b) ● = Ce(III), ▲= Ce(IV).

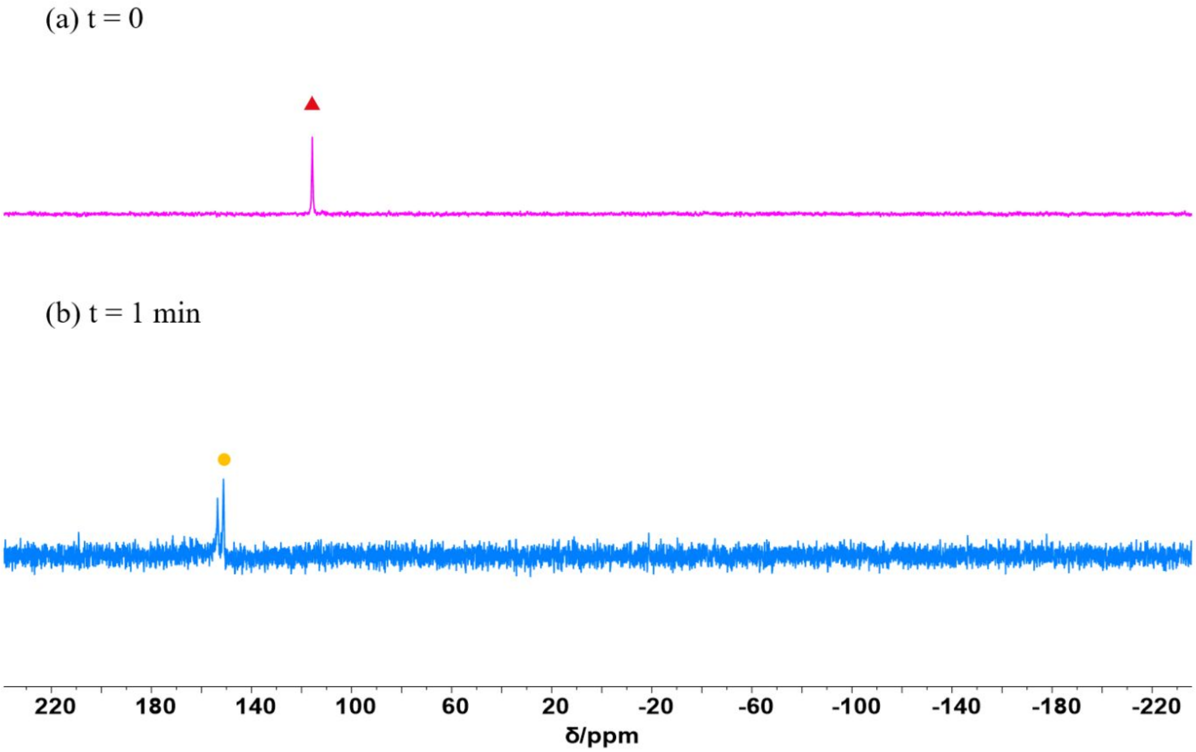

Figure S16. $^{31}P\{^{1}H\}$ NMR spectra (162 MHz, 25 ^{o}C) of $[(L_{OEt})_2CeCO_3]$ in degassed THF before (a) and after bubbling oxygen for 1 min (b). ● = Ce(III), ▲= Ce(IV).

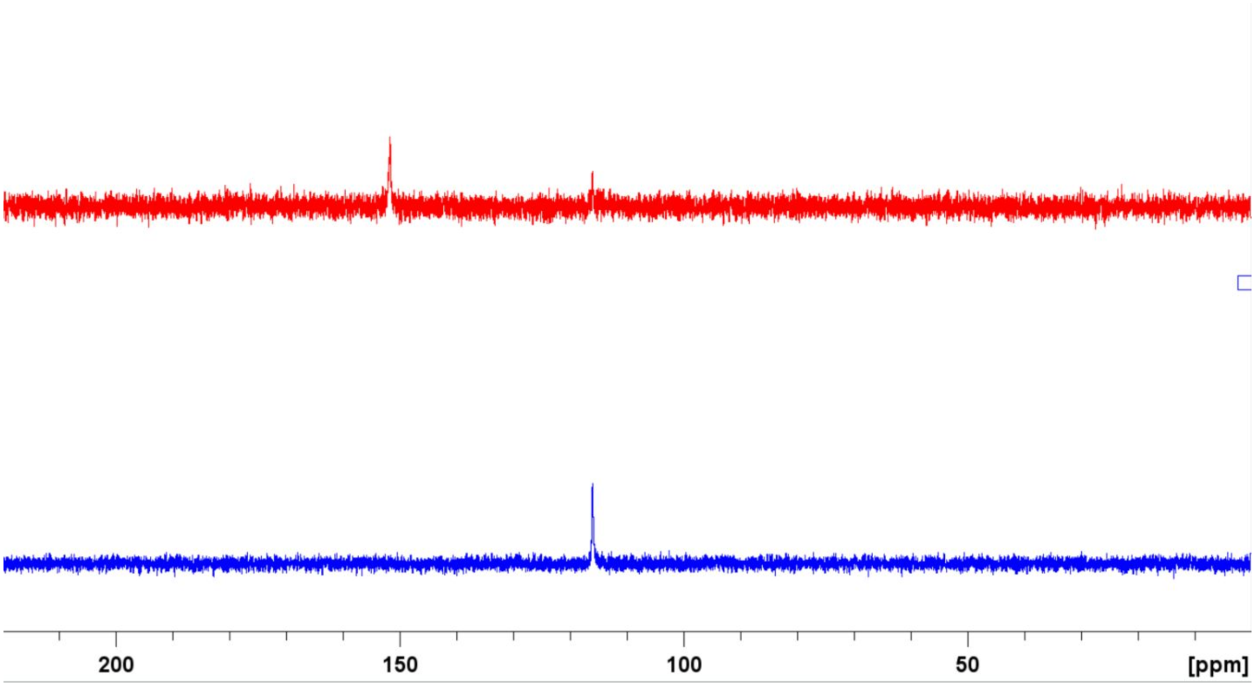

Figure S17. $^{31}P\{^1H\}$ NMR (162 MHz, 25 °C) spectra of $[Ce(L_{OEt})_2(CH_3CO_2)_2]$ in benzene before (bottom, blue line) and after irradiation with blue LED light for 30 min (top, red line). The Ce(IV)/Ce(III) ratio of the photolyzed mixture was determined to be 1:3 on the basis of the integration of the ^{31}P NMR signals.

3. MS-MS spectra

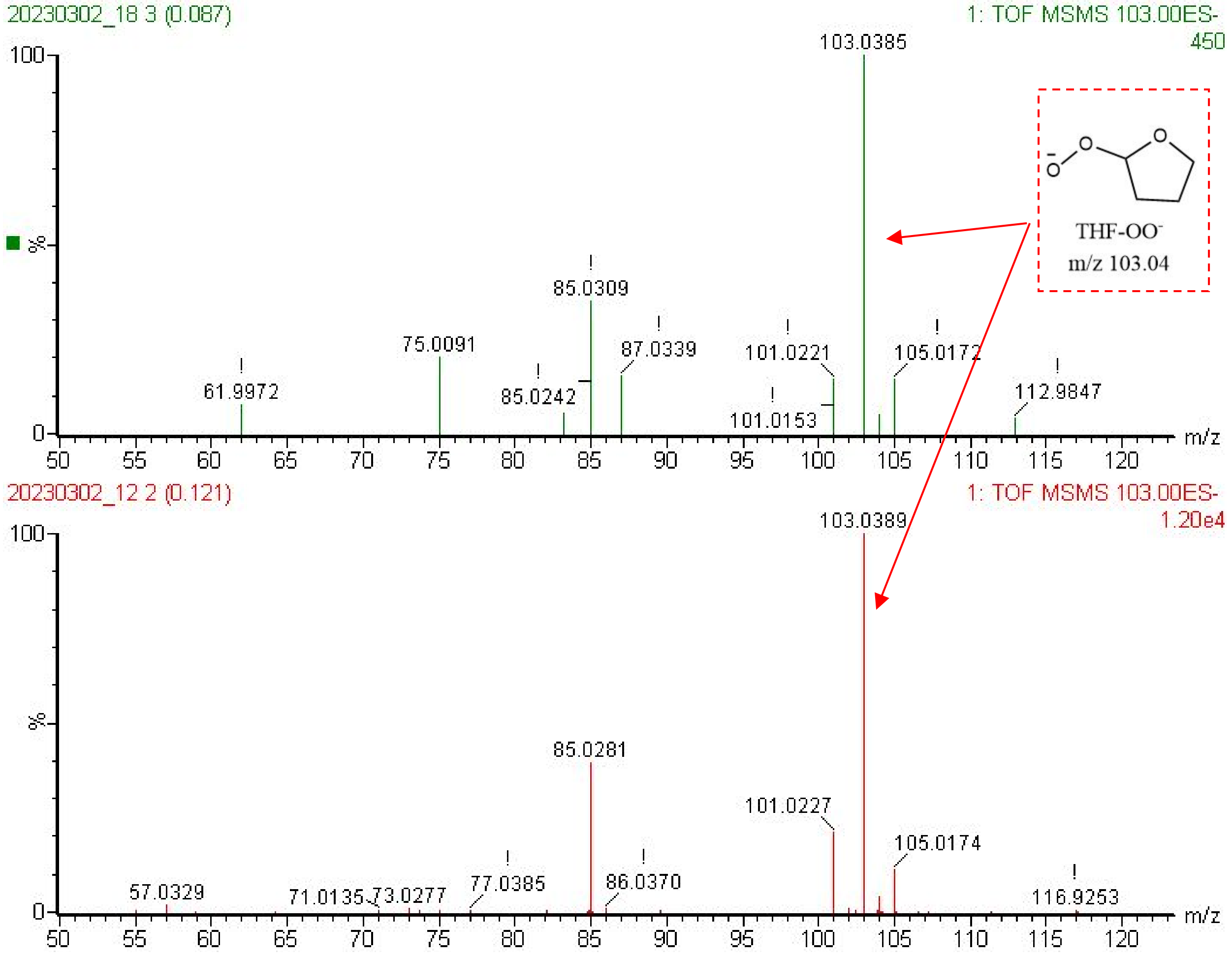

Figure S18. MS-MS spectrum of a yellow solution prepared by dissolving [Ce(L_{OEt})$_2$(CO_3)] (**1**) in distilled THF in air (top, green) and an authentic sample of tetrahydrofuran hydroperoxide synthesized according to a literature method (bottom, red).

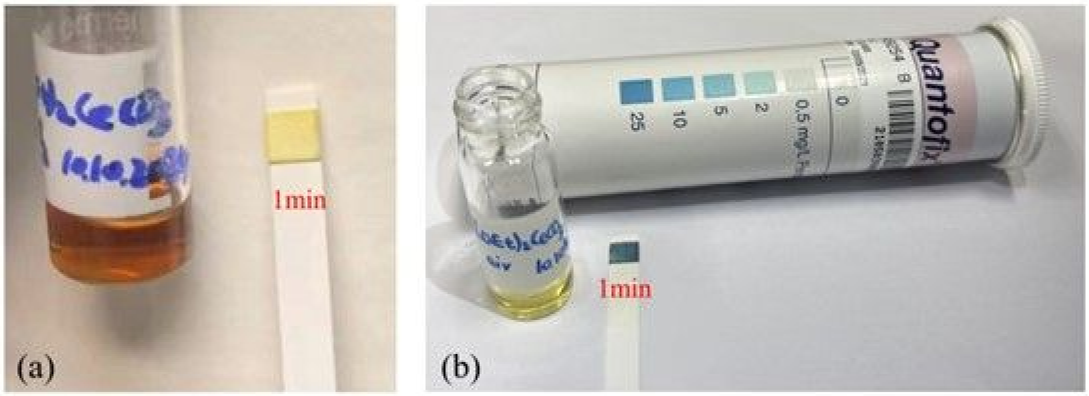

Figure S19: Peroxide test of $[(L_{OEt})_2CeCO_3]$ (5 mg) dissolved in dry THF (5 mL) under N_2 (a) and under air (b) using a Quantofix® peroxides test stick.

4. IR spectra

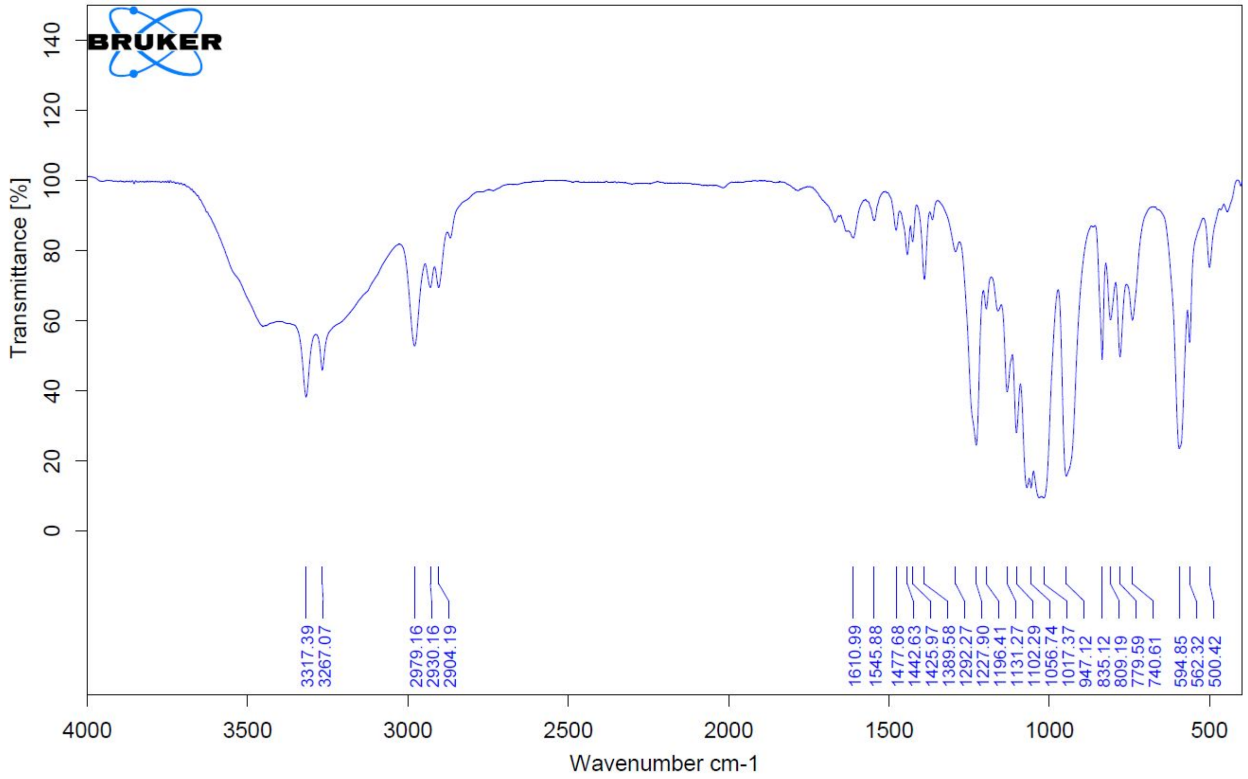

Figure S20. IR(KBr) spectrum of $[Ce(L_{OEt})_2(SO_3NH_2)_2]$ (**7**).

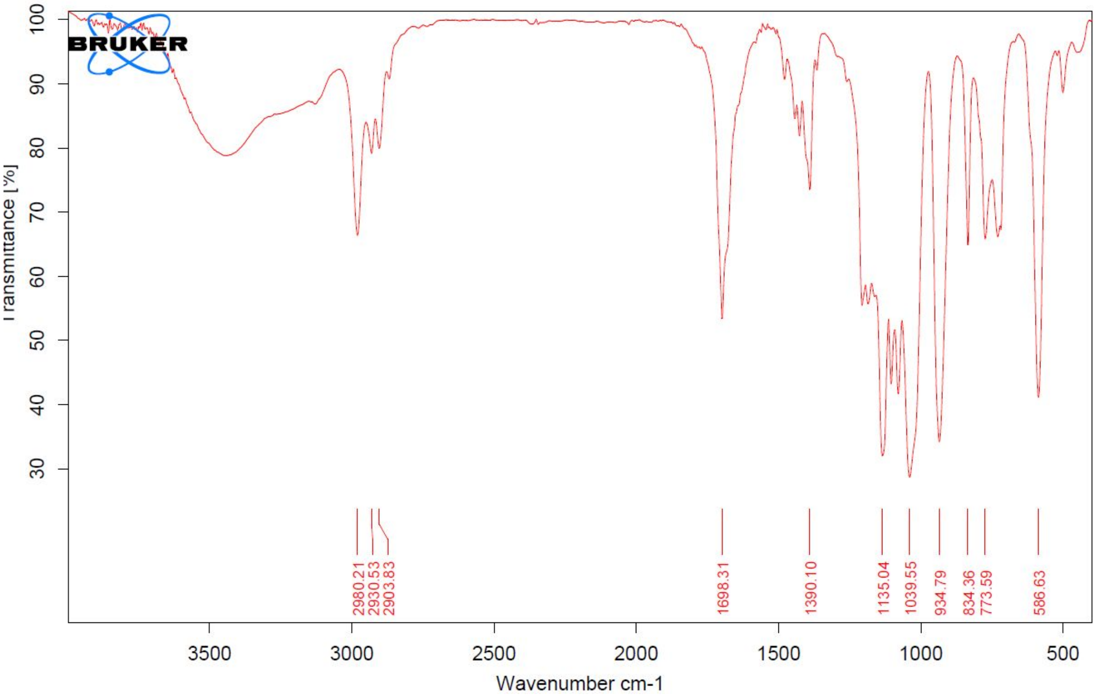

Figure S21. IR (KBr) spectrum of $[Ce(L_{OEt})_2(CONHCF_3)_2]$ (**8**)

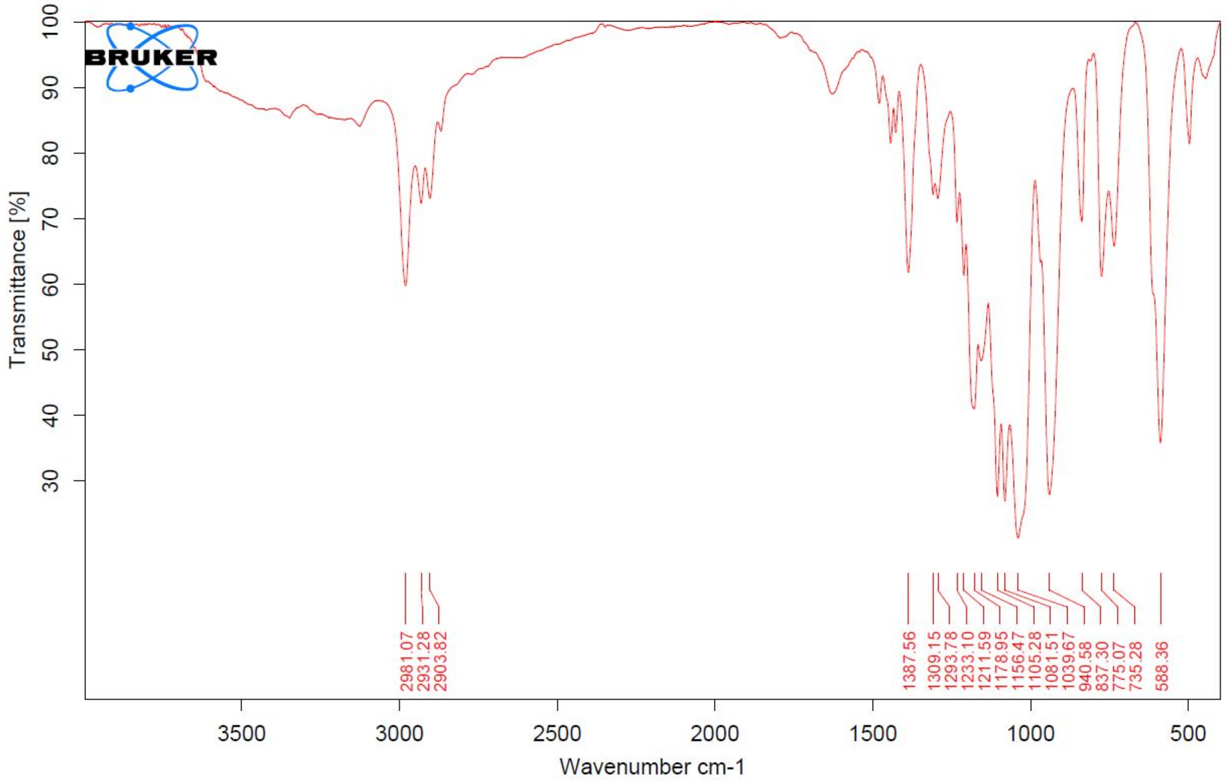

Figure S22. IR (KBr) spectrum of $[Ce(L_{OEt})_2(CF_3SO_2NH)_2]$ (**9**)
